# Novel exosomal circEGFR facilitates triple negative breast cancer autophagy via promoting TFEB nuclear trafficking and modulating miR-224-5p/ATG13/ULK1 feedback loop

**DOI:** 10.1038/s41388-024-02950-4

**Published:** 2024-01-27

**Authors:** Huachen Song, Zitong Zhao, Liying Ma, Weihong Zhao, Yi Hu, Yongmei Song

**Affiliations:** 1 Senior Department of Oncology, the Fifth Medical Center, Chinese PLA General Hospital, Beijing, 100853 China; 2 State Key Laboratory of Molecular Oncology, National Cancer Center/National Clinical Research Center for Cancer/Cancer Hospital, Chinese Academy of Medical Sciences and Peking Union Medical College, Beijing, 100021 China; 3 Department of Medical Oncology, Chinese PLA General Hospital, Beijing, 100853 China

**Keywords:** Cancer microenvironment, siRNAs, Diagnostic markers

## Abstract

Triple-negative breast cancer (TNBC) cells are in a more hypoxic and starved state than non-TNBC cells, which makes TNBC cells always maintain high autophagy levels. Emerging evidence has demonstrated that circular RNAs (circRNAs) are involved in the progress of tumorigenesis. However, the regulation and functions of autophagy-induced circRNAs in TNBC remain unclear. In our study, autophagy-responsive circRNA candidates in TNBC cells under amino acid starved were identified by RNA sequencing. The results showed that circEGFR expression was significantly upregulated in autophagic cells. Knockdown of circEGFR inhibited autophagy in TNBC cells, and circEGFR derived from exosomes induced autophagy in recipient cells in the tumor microenvironment. In vitro and in vivo functional assays identified circEGFR as an oncogenic circRNA in TNBC. Clinically, circEGFR was significantly upregulated in TNBC and was positively associated with lymph node metastasis. CircEGFR in plasma-derived exosomes was upregulated in breast cancer patients compared with healthy people. Mechanistically, circEGFR facilitated the translocation of Annexin A2 (ANXA2) toward the plasma membrane in TNBC cells, which led to the release of Transcription Factor EB (a transcription factor of autophagy-related proteins, TFEB) from ANXA2-TFEB complex, causing nuclear translocation of TFEB, thereby promoting autophagy in TNBC cells. Meanwhile, circEGFR acted as ceRNA by directly binding to miR-224-5p and inhibited the expression of miR-224-5p, which weakened the suppressive role of miR-224-5p/ATG13/ULK1 axis on autophagy. Overall, our study demonstrates the key role of circEGFR in autophagy, malignant progression, and metastasis of TNBC. These indicate circEGFR is a potential diagnosis biomarker and therapeutic target for TNBC.

## Introduction

Breast cancer is the most common and highly heterogeneous tumor in women worldwide [1]. Estrogen receptor (ER), progesterone receptor (PR), and ERBB2 receptor (HER2) expression are used to classify breast cancer into at least four subtypes (Luminal A, Luminal B, Her2-enriched, triple-negative), which laid the foundation for breast cancer classification [2]. The survival time of triple-negative breast cancer (TNBC) patients is shorter than that of other subtypes of breast cancer, and the mortality rate in the first 5 years after diagnosis is 40% [3]. Approximately 46% of TNBC patients will have distant metastasis, demonstrating its high aggressiveness. Due to its specific molecular phenotypes, TNBC is not sensitive to endocrine or molecular targeted therapy. Therefore, the new targets and treatment regimens constitute our most urgent problem to solve.

CircRNAs are the covalently closed loop RNA forms. CircRNAs are generated through a reverse splicing process, which connects the downstream 5′ splicing donor site to the upstream 3′ splicing receptor site to form the single-stranded covalently closed circular RNA molecules [4]. There are generally three types of circRNAs including exonic circRNAs (ecircRNAs), exon-intron circRNAs (EIcircRNAs), and intergenic circRNAs (ciRNAs) [5]. CircRNAs have also been found to act as miRNA sponges or decoys, protein sponges or decoys, enhancers of protein function and protein scaffolds, recruit proteins to change primary subcellular localization of proteins, and produce unique circRNA peptides, etc. [6]. Studies have indicated that circRNAs are abnormally expressed in breast cancer and involved in various biological processes of breast cancer, including proliferation, metastasis, and chemotherapy resistance [7]. CircRNAs provide a new perspective on the diagnosis and therapy of breast cancer.

Studies have shown that TNBC cells are more in a state of nutrient and oxygen deficiency than non-TNBC cells, so the autophagy levels in TNBC cells remain higher perpetually [8]. As few studies focused on how circRNAs affect TNBC progression by regulating the autophagy levels, we established an autophagy model in TNBC cells and identified circEGFR (hsa_circ_0080222) as an autophagy-associated circRNA by RNA- sequencing. CircEGFR is derived from reverse splicing of EGFR. The human EGFR family includes EGFR, HER2, ErbB3, and ErbB4. Studies have found that EGFR is overexpressed in approximately 50% of TNBC, which is associated with poor prognosis [9, 10]. When EGFR is activated by epidermal growth factor (EGF) or other ligands, it promotes autophagy, malignant growth, invasion, and metastasis of tumor cells [11, 12]. Our work suggested that circEGFR, which was upregulated in TNBC and positively correlated with lymph node metastasis, promoted autophagy and malignant phenotypes in TNBC. Moreover, exosome-derived circEGFR elevates the autophagy levels of recipient cells in the tumor microenvironment. We further elucidated that circEGFR promoted TNBC autophagy by facilitating TFEB nucleus entry through complexing with ANXA2 and sponging autophagy-related miR-224-5p. This work provides the novel insight that circEGFR functions as a critical autophagy-related oncogene in TNBC progression and exosomal circEGFR might be a promising biomarker and target for TNBC therapy.

## Results

### The expression of circEGFR is upregulated in amino acid deficiency-induced autophagy in TNBC cells

**Fig. 1:**
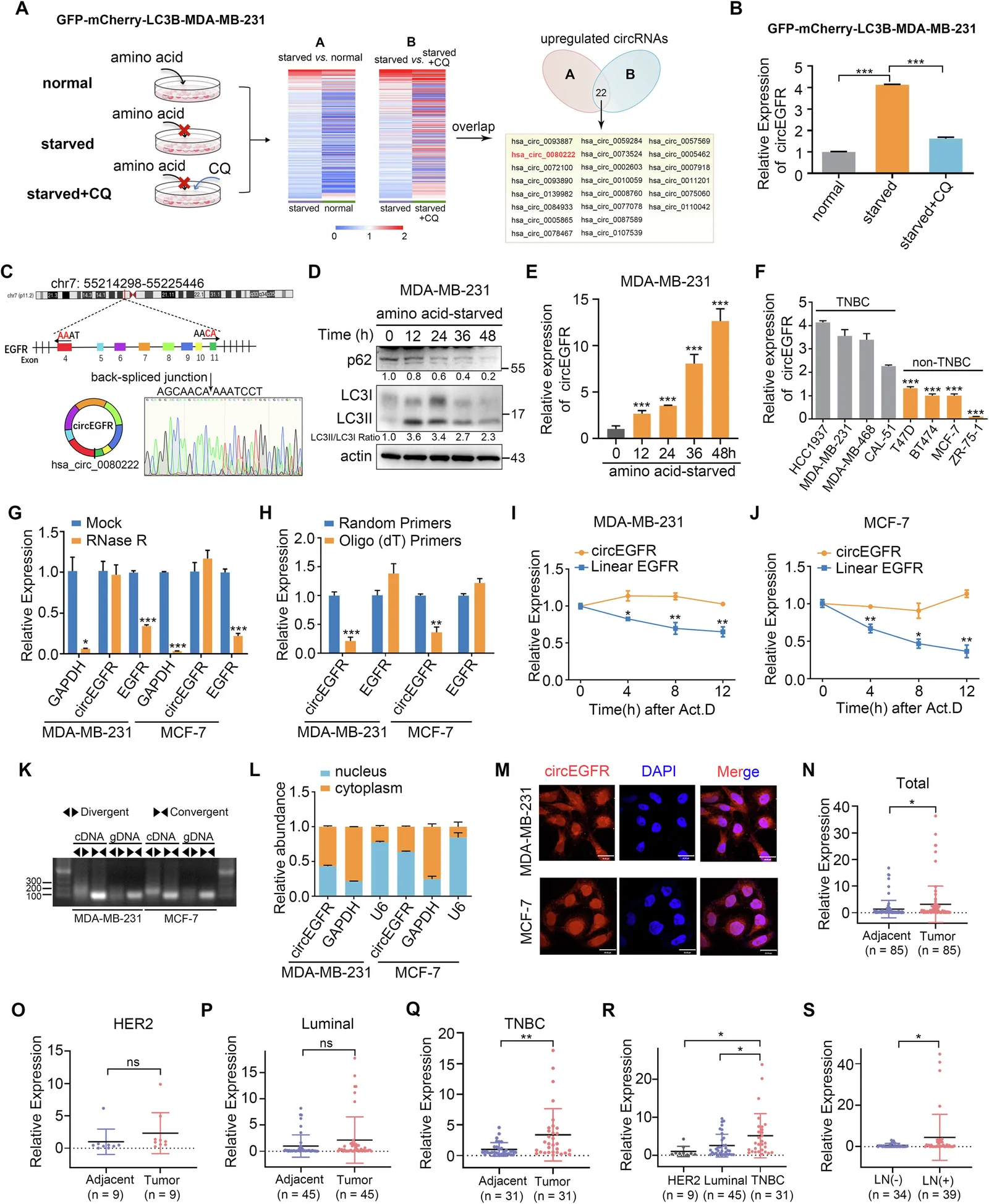
Identification of circEGFR and its expression in TNBC. **A** Establishment of intracellular autophagy-like microenvironment model and sequencing strategy. **B** The expression of circEGFR in normal, starved, and CQ-treated starved GFP-mCherry-LC3B-labeled MDA-MB-231 cells. **C** Schematic illustration indicating circularization of circEGFR generated from EGFR and the validation of back-splicing site with Sanger sequencing. **D** The expression of p62 and LC3II in amino acid-starved MDA-MB-231 cells at 0, 12, 24, 36, and 48 h. **E** The expression of circEGFR during the period of autophagy in MDA-MB-231 cells. **F** The expression of circEGFR in TNBC cells and non-TNBC cells. **G** qPCR determined the expression of circEGFR and linear transcript EGFR with or without RNase R treatment in MDA-MB-231 and MCF-7 cells. **H** qPCR analysis of circEGFR and linear transcript EGFR using random primers or oligo-dT primers in MDA-MB-231 and MCF-7 cells. Analysis of circEGFR and EGFR mRNA stability with actinomycin D treatment in **I** MDA-MB-231 and **J** MCF-7 cells. **K** PCR assays and agarose gel electrophoresis assay showed the amplifcation of circEGFR using divergent and convergent primers in the cDNA and gDNA of MDA-MB-231 and MCF-7 cells. **L** CircEGFR levels in the cytoplasm or nucleus of MDA-MB-231 and MCF-7 cells. **M** The subcellular localization of circEGFR in MDA-MB-231 and MCF-7 cells conducted by FISH. Scale bar, 30 μm. qPCR showed the expression of circEGFR in tumor tissues and paired adjacent tissues of (**N**) all (*n* = 85), **O** Her2-type (*n* = 9), **P** Luminal-type (*n* = 45), and **Q** TNBC patients (*n* = 31). **R** qPCR showed the expression of circEGFR in with Her2-type, Luminal-type, and TNBC tissues. **S** CircEGFR levels in the non-lymph node (*n* = 34) and lymph node metastasis tissues (*n* = 39). Data are shown as the means ± SD. ns, *P* > 0.05; *, *P* < 0.05; **, *P* < 0.01; ***, *P* < 0.001.

To identify autophagy-associated circRNAs in TNBC cells, GFP-mCherry-LC3B-labeled MDA-MB-231 cells were cultured with the amino acid-free DMEM (starved) for 48 h to achieve an autophagic microenvironment. We administered amino acid-free DMEM containing 20 μM Chloroquine (CQ, an autophagy inhibitor) after seeding GFP-mCherry-LC3B-labeled MDA-MB-231 cells for 24 h. First, the autophagy levels of the three groups of cells was determined. The results showed that the autophagic flux of the starved cells was significantly increased compared with the normal cells and CQ-treated starved cells (Fig. S1A, S1B). p62 was specifically degraded in intact autophagy [13]. In the presence of CQ, the accumulation of LC3II indicated that autophagic flux was blocked. Western Blot results revealed that p62 expression was decreased and LC3II expression was increased in the starved cells. (Fig. S1C). The above data indicated that the autophagy levels of the starved cells were significantly elevated. Then we performed the RNA- sequencing of the three groups (Fig. 1A, GSA: HRA005935). In RNA sequencing analysis, 22 circRNA candidates were screened according to the following standards: (i) the starved group *vs*. the normal group, fold change >5; (ii) the starved group *vs*. the CQ-treated starved group, fold change >5; (iii) High conservation; (iv) the length <1000 nt. Then we found that only circEGFR (hsa_circ_0080222) was substantially higher in starved cells than in normal cells and CQ-treated starved cells (Fig. S1D and Fig. 1B). We selected circEGFR as the object for further research. CircEGFR is located on chromosome 7 and is formed by reverse splicing of EGFR. It is an ecircRNA with 874 nt in length and composed of eight exons. We validated the back-splicing site of circEGFR by Sanger sequencing (Fig. 1C). MDA-MB-231 cells exposed to starvation at different time points were collected, and we found that MDA-MB-231 cells occurred obvious autophagy within 48 h by Western Blot (Fig. 1D). qPCR results showed that circEGFR expression was significantly increased over time (Fig. 1E). These data reveal that circEGFR could be induced by the autophagic environment of TNBC cells.

The qPCR results revealed that circEGFR expression was markedly higher in TNBC cell lines than in non-TNBC cell lines (Fig. 1F). Next, we conducted circRNA identification of circEGFR. In RNase R digestion experiment, circEGFR was more resistant to RNase R than the linear transcript EGFR in MDA-MB-231 and MCF-7 cells (Fig. 1G). Since circRNA cannot be reverse transcribed by oligo primers, we found that oligo primers could reverse transcribed EGFR, but not circEGFR (Fig. 1H). To further determine the stability of circEGFR, cells were treated with actinomycin D. The results revealed that actinomycin D treatment led a longer half-life of circEGFR than the linear transcript mRNA, indicating that circEGFR was more stable (Fig. 1I and J). The cDNA and genomic DNA (gDNA) were amplified by designed divergent or convergent primers. The divergent primers could amplify circEGFR in cDNA, but not in gDNA (Fig. 1K). Moreover, the qPCR analysis and FISH results of RNA in nucleus and cytoplasm of MDA-MB-231 and MCF-7 cells displayed that circEGFR was mainly distributed in the cytoplasm (Fig. 1L, M).

The expression of circEGFR was higher in breast cancer tissues than that in paired paracancerous tissues (Fig. 1N). Although there was no significant difference in the expression of circEGFR in tumor tissues compared with the adjacent tissues of HER2 and Luminal subtypes, there is an increasing trend (Fig. 1O, P). The expression of circEGFR was significantly overexpressed in tumor tissues compared with paired adjacent tissues of TNBC patients (Fig. 1Q). Compared with HER2-type and Luminal-type tissues, the expression of circEGFR was upregulated in TNBC-type tissues (Fig. 1R). CircEGFR expression in the tissues with lymph node metastasis was higher than that in non-metastatic tissues (Fig. 1S). The above data suggest that circEGFR is a stable circRNA mainly located in the cytoplasm, and participates in the autophagy process, as well as plays the role of oncogene to promote the progress of TNBC.

### CircEGFR promotes TNBC autophagy and malignant phenotypes both in vitro and in vivo

**Fig. 2:**
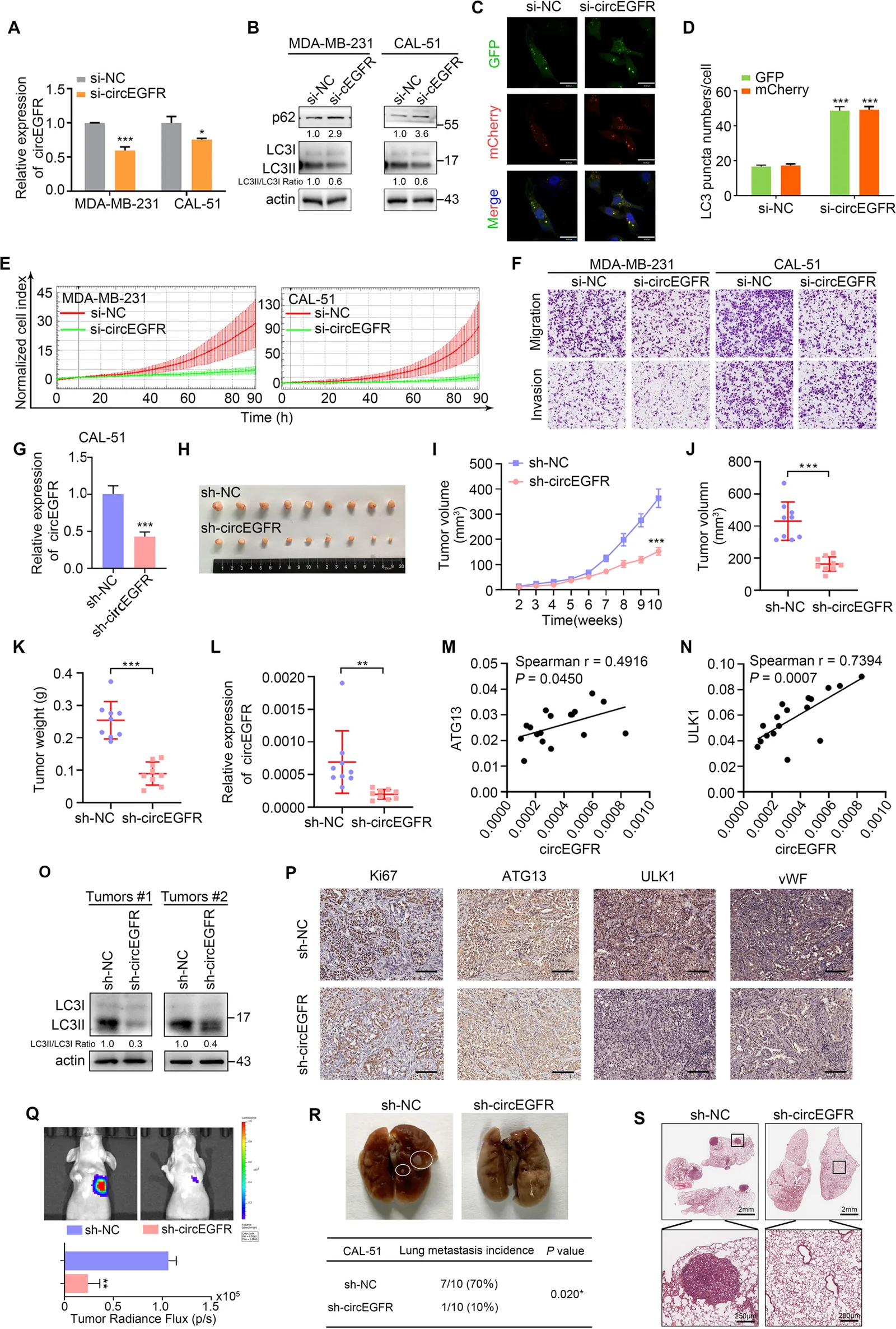
CircEGFR promotes TNBC autophagy and malignant phenotypes both in vitro and in vivo. **A** The expression of circEGFR in MDA-MB-231 and CAL-51 cells transfected with si-circEGFR or si-NC. **B** Western Blot results of p62 and LC3II in MDA-MB-231 and CAL-51 cells transfected with si-circEGFR or si-NC. **C** Representative images of autophagy levels in GFP-mCherry-LC3B-labeled MDA-MB-231 cells transfected with si-circEGFR or si-NC were observed by IF. Scale bar, 30 μm. **D** Quantitative analysis of LC3 puncta numbers in (**C**). **E** Cell proliferation analysis in MDA-MB-231 and CAL-51 cells. **F** Cell migration and invasion analysis in MDA-MB-231 and CAL-51 cells. Original magnification, ×100. **G** The verification of stable circEGFR knockdown by qPCR. **H** The image of xenograft tumors. **I** The tumor volume was measured weekly using the formula (L × W^2^)/2. Analysis of the growth of tumor in (**J**) volume and (**K**) weight. **L** The expression of circEGFR in xenograft tumors. **M** The correlation analysis between circEGFR and ATG13 expression in xenograft tumors. **N** The correlation analysis between circEGFR and ULK1 expression in xenograft tumors. **O** Western Blot results of LC3II in xenograft tumors. **P** The expression of Ki67, ATG13, ULK1, and vWF in xenograft tumors was determined by IHC analysis. Scale bar, 100 μm. **Q** The luciferase images of lung metastases. **R** Representative photographs of the lung tissues and the rate of lung metastasis. The statistical difference was assessed with Chi-square test and Fisher’s exact test, *, *P* < 0.05. **S** HE images of metastatic loci in lungs in caudal vein metastasis model. Scale bar, 2 mm and 250 μm (inset), respectively. Data are shown as the means ± SD. *, *P* < 0.05; **, *P* < 0.01; ***, *P* < 0.001.

In order to determine the autophagy-related role of circEGFR in TNBC, we selected MDA-MB-231 and CAL-51 cells with relatively higher expression of circEGFR for knockdown, and designed siRNA to knockdown circEGFR (Fig. 2A). Western Blot results showed that knockdown of circEGFR increased the expression of p62 and decreased LC3II expression (Fig. 2B). The decrease of circEGFR expression reduced the autophagic flux of TNBC cells (Fig. 2C, D). The overexpression plasmids were transfected into non-TNBC cells including MCF-7 and T47D cells with relatively lower expression of circEGFR (Fig. S2A). We found that overexpression of circEGFR reduced p62 expression, increased LC3II expression (Fig. S2B), and increased autophagic flux (Fig. S2C, S2D). In addition, we further explored the oncogene function of circEGFR in TNBC cells. In MDA-MB-231 and CAL-51 cells, knockdown of circEGFR inhibited proliferation (Fig. 2E), migration, invasion (Fig. 2F and Fig. S2E), colony formation (Fig. S2F), and promoted G1 phase arrest (Fig. S2G, S2H) and cell apoptosis (Fig. S2I, S2J). Overexpression of circEGFR exerted the opposite effect (Fig. S2K–S2P). These findings support that circEGFR could increase the autophagy levels and promote the formation of malignant phenotypes in TNBC cells.

To investigate the loss-of-function effects of circEGFR in vivo, we constructed CAL-51 cells that stably circEGFR knockdown (sh-circEGFR) and negative control cells (sh-NC) (Fig. 2G). The tumor cells were subcutaneously injected in the mammary fat pad of BALB/c nude mice. Ten weeks later, the mice were euthanized and the tumor masses were isolated. We found that downregulation of circEGFR suppressed tumor size (Fig. 2H–J) and tumor weight (Fig. 2K). The qPCR results revealed that circEGFR expression was significantly decreased in sh-circEGFR tumors (Fig. 2L). Western Blot results showed that LC3II expression was reduced in sh-circEGFR xenograft tumors (Fig. 2O). By IHC, the expression of Ki67 was decreased in sh-circEGFR tumors compared with the control tumors (Fig. 2P). We established the lung metastases model by injecting sh-circEGFR or sh-NC cells in the tail veins of nude mice. The bioluminescence images showed that downregulation of circEGFR decreased the fluorescence intensity of the lungs compared with the control group (Fig. 2Q). The results showed that knockdown of circEGFR reduced the rate of lung metastasis in mice (Fig. 2R, S). The above data imply that circEGFR promotes the progression of TNBC in vivo.

### Exosome-derived circEGFR promotes autophagy of recipient cells in tumor microenvironment

**Fig. 3:**
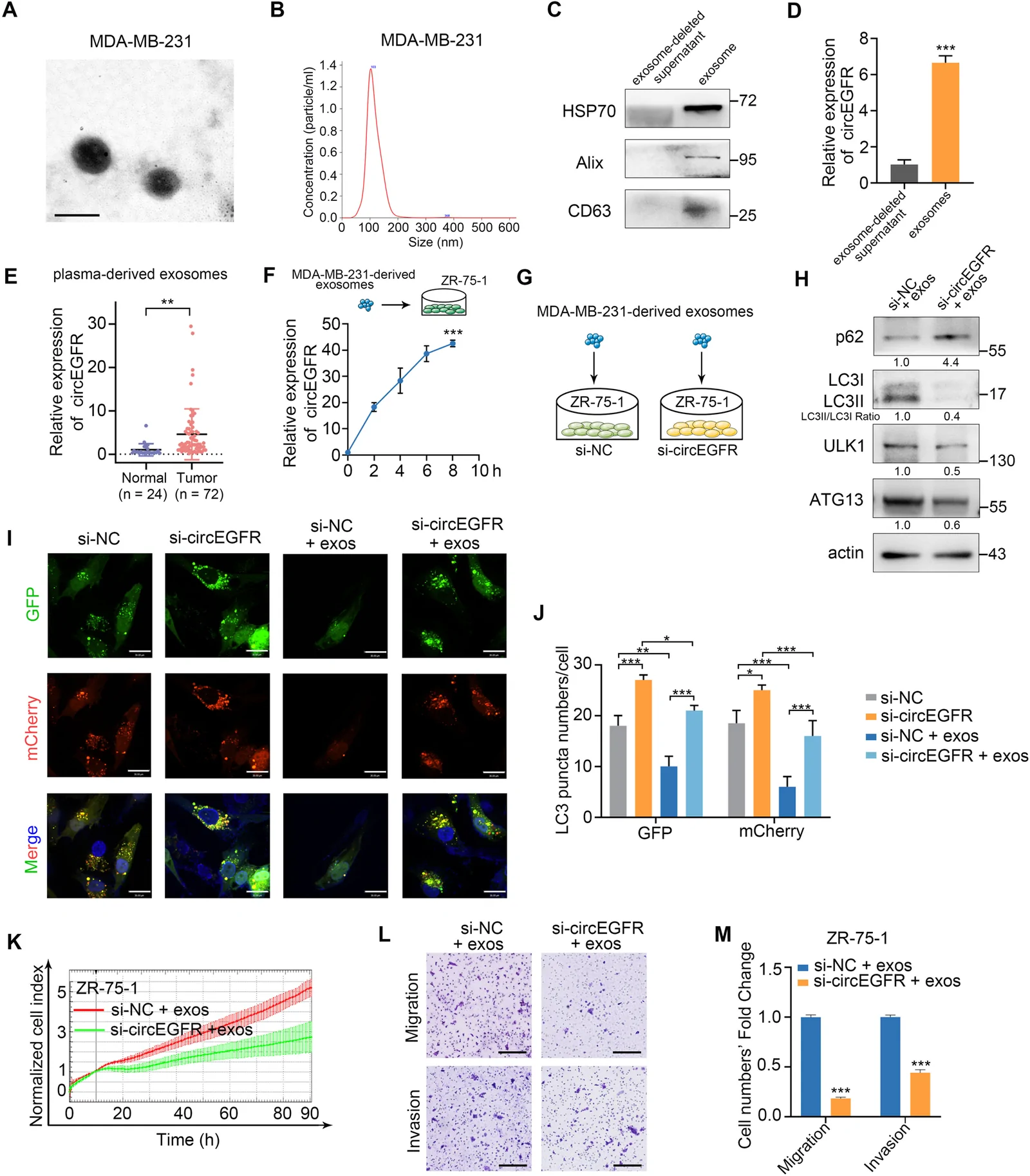
Exosome-derived circEGFR promotes autophagy of recipient cells in tumor microenvironment. **A** Representative images of exosomes derived from MDA-MB-231 cells were observed by transmission electron microscope. Scale bar, 100 nm. **B** Nanosight analysis of exosomes derived from MDA-MB-231 cells. **C** The expression of HSP70, Alix, and CD63 in exosome-deleted supernatant and exosomes was measured by Western Blot. **D** The expression of circEGFR in exosome-deleted supernatant and exosomes. **E** The expression of circEGFR in exosomes derived from the plasma samples of healthy people (*n* = 24) and breast cancer patients (*n* = 72) by qPCR. **F** Time course of circEGFR expression in ZR-75-1 cells following treatment in the absence or presence of MDA-MB-231-derived exosomes. **G** MDA-MB-231-derived exosomes were added to ZR-75-1 cells transfected with si-circEGFR or si-NC. **H** ZR-75-1 cells transfected with si-circEGFR or si-NC were treated with or without MDA-MB-231-derived exosomes and then Western Blot was used to determine the expression of autophagy-associated protein, ULK1, and ATG13. **I** GFP-mCherry-LC3B-labeled MDA-MB-231 cells transfected with si-circEGFR or si-NC were treated with or without MDA-MB-231-derived exosomes and then were observed by IF to detect autophagy levels. Scale bar, 30 μm. **J** Quantitative analysis of LC3 puncta numbers in (**I**). ZR-75-1 cells transfected with si-circEGFR or si-NC were treated with or without MDA-MB-231-derived exosomes and then (**K**) cell proliferation, (**L**) (top) migration, and (**L**) (bottom) invasion was determined. (Original magnification, ×100). **M** Quantitative analysis of cell migration and invasion in (**L**). Data are shown as the means ± SD. *, *P* < 0.05; **, *P* < 0.01; ***, *P* < 0.001.

In view of the characteristics of circRNAs, including higher abundance than linear transcripts in exosomes and their structural stability, we investigated the presence and function of circEGFR in exosomes derived from TNBC cells. First, we observed the structure of MDA-MB-231-derived exosomes by transmission electron microscope (TEM). The structure was found to be a disk-like vesicle with lipid bilayer (Fig. 3A). The extracted exosomes were confirmed to be enriched at 50-150 nm in diameter by Nanosight analysis (Fig. 3B). We verified the expression of exosome marker HSP70, Alix, and CD63 in the extracted exosomes by Western Blot (Fig. 3C). Based on the above data, the extracted exosomes from MDA-MB-231 cells were identified. The qPCR assay was performed to determine the abundance of circEGFR in MDA-MB-231-derived exosomes (Fig. 3D). The plasma from 24 healthy people and 72 breast cancer patients were collected and the exosomes were extracted. We found that circEGFR was highly expressed in breast cancer patients compared with healthy people (Fig. 3E).

To further substantiate the effect of exosomes secreted by TNBC cells with high circEGFR expression on other tumor cells in the tumor microenvironment, we added the extracted exosomes from MDA-MB-231 cells to ZR-75-1 cells. The expression of circEGFR in ZR-75-1 cells significantly increased over time, reaching peak expression 8 h after treatment (Fig. 3F). Due to the low abundance of circEGFR in ZR-75-1 cells, transfection of si-circEGFR to knockdown circEGFR had no significant effect on the autophagy phenotypes. Therefore, the effect of circEGFR in MDA-MB-231-derived exosomes on the autophagy phenotypes could be observed (Fig. 3G). The results showed that MDA-MB-231-derived exosomes inhibited the expression of LC3II and promoted p62 expression in ZR-75-1 cells transfected with si-circEGFR (Fig. 3H). The autophagic flux of GFP-mCherry-LC3B-labeled MDA-MB-231 cells was elevated after treatment with exosomes derived from MDA-MB-231. Compared with the control group (si-NC + exos), the autophagic flux of the cells with decreased circEGFR expression was diminished after exosome treatment (si-circEGFR + exos). Moreover, the changes of autophagic flux between exosome-treated cells were more significant than between the cells untreated with exosomes (Fig. 3I, J). Similarly, we found that MDA-MB-231-derived exosomes inhibited malignant phenotypes of recipient cells including proliferation, migration, and invasion (Fig. 3K–M). These data suggest that exosome-derived circEGFR could elevate the autophagy levels and malignant phenotypes of other breast cancer cells with a low abundance of circEGFR in the tumor microenvironment.

### ANXA2 binds to circEGFR in TNBC cells

**Fig. 4:**
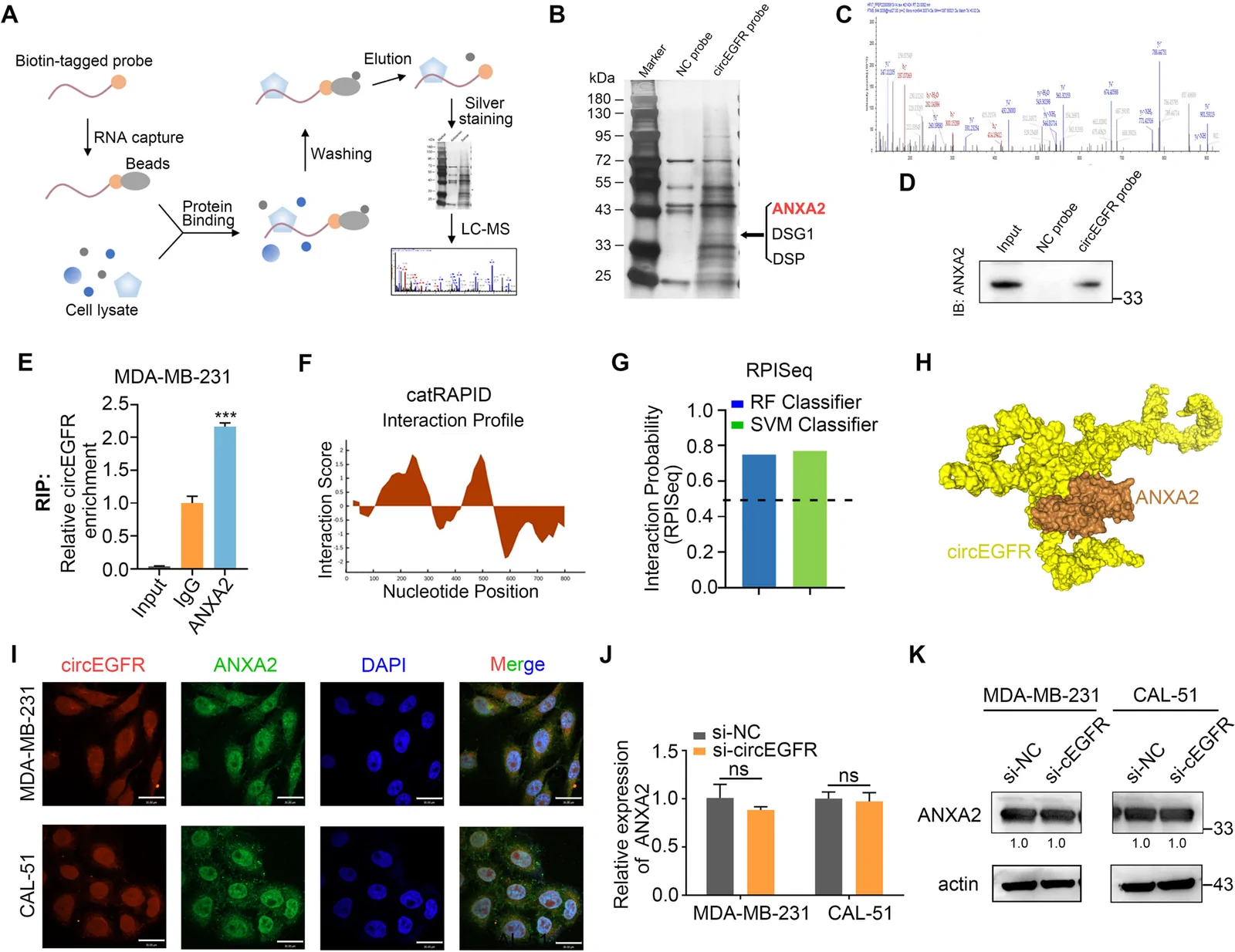
ANXA2 binds to circEGFR in TNBC cells. **A** The workflow diagram of protein isolation by RNA pulldown assays used to identify potential circEGFR-binding proteins. **B** Silver staining showed different positive bands in circEGFR probe group compared with NC probe group. The proteins enriched with circEGFR were identified by mass spectrometry analysis, and ANXA2 was selected as the research object related to autophagy. **C** Mass spectrometry results of the ANXA2 peptides pulled down by circEGFR probes. **D** The interaction between circEGFR and ANXA2 in MDA-MB-231 cells was confirmed by Western Blot. **E** RIP assay was performed in MDA-MB-231 cells using ANXA2 antibody. The enrichment of circEGFR was determined by qPCR analysis. **F** The binding sites between circEGFR and ANXA2 were predicted in catRAPID database. **G** The interaction possibility of circEGFR and ANXA2 was detected in RPISeq database. **H** The schematic diagram showed that circEGFR bound to ANXA2. **I** The colocalization of circEGFR and ANXA2 was observed by IF and FISH. Scale bar, 30 μm. **J** The mRNA expression of ANXA2 in MDA-MB-231 and CAL-51 cells transfected with si-circEGFR or si-NC was measured by qPCR. **K** The protein expression of ANXA2 in MDA-MB-231 and CAL-51 cells transfected with si-circEGFR or si-NC was measured by Western Blot. Data are shown as the means ± SD. ns, *P* > 0.05; ***, *P* < 0.001.

We further explored the molecular mechanism of circEGFR promoting the autophagy process in TNBC cells. There is growing evidence that circRNAs are involved in the oncogenesis and development of tumors through binding proteins [14]. We performed RNA pulldown by incubating Biotin-labeled circEGFR probes with lysates of MDA-MB-231 cells and analyzed the positive band in circEGFR probe group by mass spectrometry (Fig. 4A). We found ANXA2, which has been reported to be involved in autophagy [15, 16], among the candidate proteins, so we selected ANXA2 as the further focus of our work (Fig. 4B). Mass spectrometry and RNA pulldown results verified the binding of circEGFR with ANXA2 (Fig. 4C, D). The RIP results also revealed that circEGFR was significantly enriched in the anti-ANXA2 group compared with the IgG group (Fig. 4E). CatRAPID database analysis (http://s.tartaglialab.com/page/catrapid_group) showed a strong trend of ANXA2 binding with circEGFR at 276-327 nt and 474-525 nt (Fig. 4F). The analysis of the possibility of circRNA binding with proteins in RPISeq database (http://pridb.gdcb.iastate.edu/RPISeq/) predicted circEGFR could interact with ANXA2 (Fig. 4G). We used HDOCK SERVER (http://hdock.phys.hust.edu.cn/) to analyze the interaction between circEGFR and ANXA2. During this process, the secondary structure of circEGFR was predicted on the RNAfold Web server (http://rna.tbi.univie.ac.at/cgi-bin/RNAWebSuite/RNAfold.cgi). Then the secondary structure obtained was analyzed to further predict the tertiary structure of 3dRNA (http://biophy.hust.edu.cn/3dRNA) [17]. In addition, we obtained the tertiary structure of ANXA2 from Protein Data Bank (https://www.wwpdb.org/). Subsequently, these information was imported into HDOCK SERVER [18]. The prediction result of the interaction between circEGFR and ANXA2 was displayed in Fig. 4H. The colocalization of circEGFR and ANXA2 was observed by IF in MDA-MB-231 and CAL-51 cells (Fig. 4I). The above data elucidate that circEGFR could interact with ANXA2. Next, we investigated how circEGFR affected ANXA2 in facilitating autophagy. The qPCR and Western Blot results showed that circEGFR did not affect mRNA and protein expression of ANXA2 (Fig. 4J, K). We speculate that circEGFR might lead to localization change of ANXA2 in TNBC cells.

### CircEGFR-regulated TFEB nucleus translocation promotes autophagy and ANXA2 plasma membrane trafficking facilitates angiogenesis in TNBC cells

We observed the localization of ANXA2 in MDA-MB-231 cells transfected with circEGFR plasmid and si-circEGFR by IF. The results showed that overexpression of circEGFR facilitated the translocation of ANXA2 toward the plasma membrane (PM) (Fig. S3A). Knockdown of circEGFR inhibited the translocation of ANXA2 toward the PM (Fig. S3B). When circEGFR expression was elevated, circEGFR was still localized mainly in the cytoplasm and its binding protein ANXA2 was translocated to the PM by the regulation of circEGFR (Fig. S3C). Previous results have shown that circEGFR increased autophagy levels in TNBC cells. We investigate whether elevated autophagy levels induced by circEGFR could promote the translocation of ANXA2 toward the PM. The results displayed that ANXA2 translocated toward the PM in starved MDA-MB-231 cells (Fig. S3D). It suggested that increased autophagy levels facilitates the translocation of ANXA2 toward the PM. These results elucidated that the translocation of ANXA2 toward the PM depends on circEGFR-induced autophagy.

**Fig. 5:**
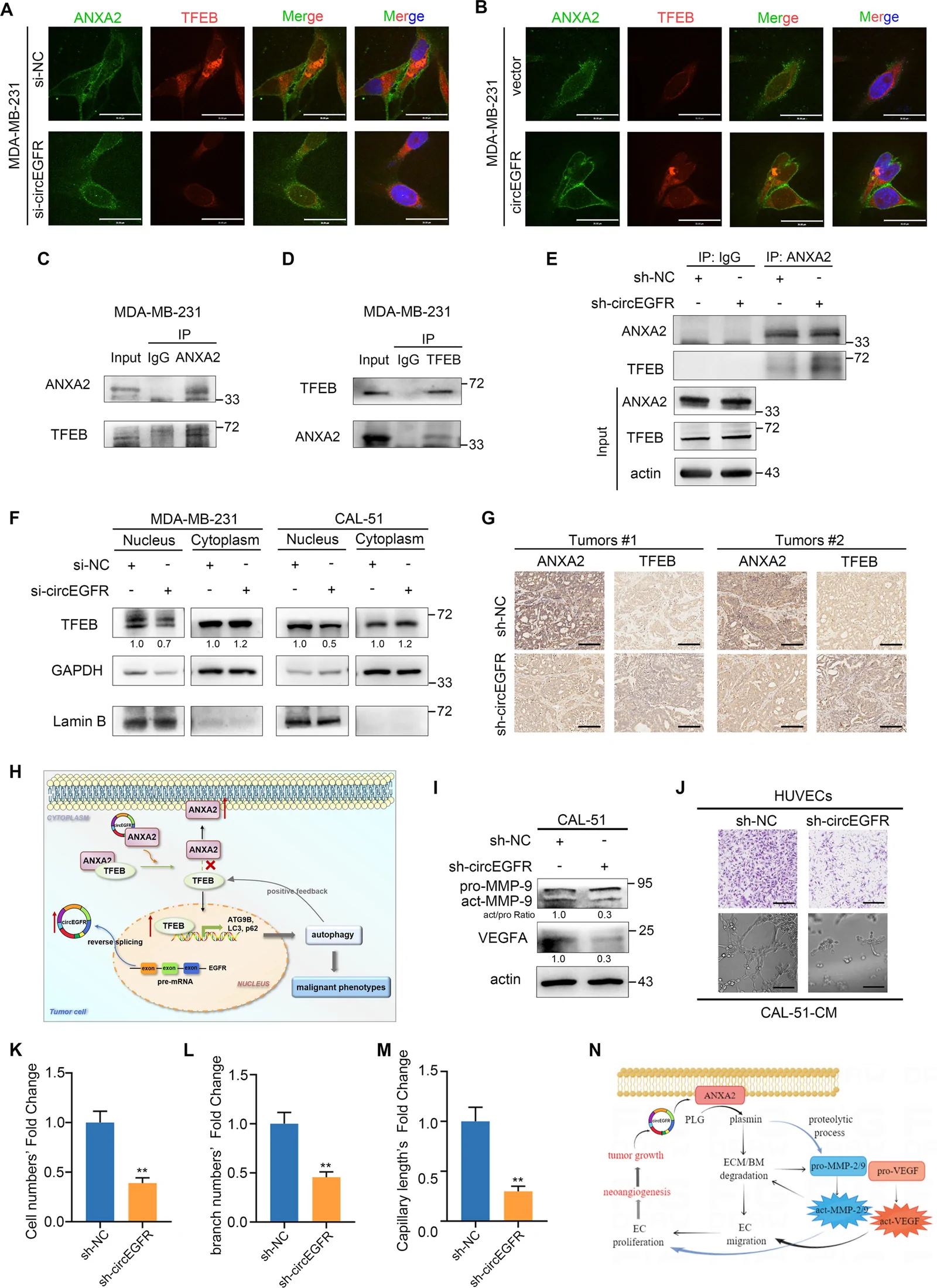
CircEGFR-regulated TFEB nucleus translocation promotes autophagy and ANXA2 plasma membrane trafficking facilitates angiogenesis in TNBC. **A** IF was performed in MDA-MB-231 cells transfected with si-circEGFR or si-NC using ANXA2 and TFEB antibodies. Scale bar, 30 μm. **B** IF was performed in MDA-MB-231 cells expressing circEGFR or vector using ANXA2 and TFEB antibodies. Scale bar, 30 μm. **C** Co-IP assay was performed in MDA-MB-231 cells using IgG and ANXA2 antibodies. **D** Co-IP assay was performed in MDA-MB-231 cells using IgG and TFEB antibodies. **E** Co-IP assay was performed in CAL-51 cells stably knocked down circEGFR using IgG and ANXA2 antibodies. **F** MDA-MB-231 and CAL-51 cells were transfected with si-circEGFR or si-NC, and then subcellular fractionation assay was used to detect the translocation of TFEB. **G** IHC assay was performed in xenograft tumors using ANXA2 and TFEB antibodies. Scale bar, 100 μm. **H** The schematic diagram of circEGFR/ANXA2/TFEB axis in TNBC cells. **I** The expression of active MMP-9 and VEGFA in CAL-51 cells stably knocked down circEGFR was measured by Western Blot. **J** Representative images of HUVECs cultured with conditioned medium from CAL-51 cells stably knocked down circEGFR were measured by migration assay (original magnification, ×100) and matrigel tube formation assay (scale bar: 2 mm). **K** Quantitative analysis of cell numbers in migration assay. **L** Quantitative analysis of branch numbers in matrigel tube formation assay. **M** Quantitative analysis of capillary length in matrigel tube formation assay. **N** The schematic diagram of the relationship between ANXA2 on the plasma membrane induced by circEGFR and angiogenesis. Data are shown as the means ± SD. **, *P* < 0.01.

It has been found that ANXA2 binds to TFEB to form a complex that blocks TFEB from entering into the nucleus to perform its key transcription factor of autophagy [19, 20]. TFEB could act as a transcription factor for autophagy-associated proteins including ATG9B, LC3, and p62 [21–23]. Therefore, we hypothesized that the translocation of ANXA2 toward the PM induced by circEGFR might be involved in the autophagy process of TNBC cells. Then we examined the effects of circEGFR on ANXA2-TFEB interaction. Knockdown of circEGFR promoted colocalization of ANXA2 and TFEB in the cytoplasm of MDA-MB-231 and CAL-51 cells (Fig. 5A and Fig. S3E). Overexpression of circEGFR facilitated the translocation of ANXA2 toward the PM and TFEB toward the nucleus (Fig. 5B and Fig. S3E). As reported previously, TFEB is negatively regulated by mTORC1 and is released upon starvation [24]. IF results showed that TFEB underwent increased nucleus translocation when cells were exposed to starvation (Fig. S3D). The coIP results revealed that ANXA2 interacted with TFEB in MDA-MB-231 cells (Fig. 5C, D). The stably knockdown of circEGFR promoted enhancement of interaction between ANXA2 and TFEB by coIP (Fig. 5E). Similarly, subcellular fractionation assay displayed that knockdown of circEGFR decreased TFEB expression in the nucleus of MDA-MB-231 and CAL-51 cells (Fig. 5F). Moreover, the translocation of ANXA2-TFEB was observed in xenograft tumors (Fig. 5G). These data suggest that circEGFR elevates autophagy levels in TNBC cells by competitively binding to ANXA2, separating ANXA2-TFEB complex, blocking the interaction between ANXA2 and TFEB in the cytoplasm, and facilitating the translocation of ANXA2 toward the PM and TFEB toward the nucleus (Fig. 5H). The above data also reveal a positive feedback loop, namely, the high expression of circEGFR in TNBC cells increases the autophagy levels, thereby promoting the entry of TFEB into the nucleus, which increases the levels of autophagy in TNBC cells, suggesting that circEGFR is a critical oncogene in TNBC.

Studies have found that ANXA2 located on the PM could act as a receptor for plasminogen (PLG) and convert PLG to plasmin, which promotes ECM/BM degradation, and activation of MMP-2/9 and VEGF, thus facilitating the proliferation and migration of a large number of endothelial cells (ECs), promoting angiogenesis, and further accelerating the malignant progression of tumors [25, 26]. Since circEGFR-induced the translocation of ANXA2 toward the PM, we investigated whether circEGFR contributes to angiogenesis in TNBC cells. We found that circEGFR knockdown inhibited the expression of active MMP-9 and VEGFA (Fig. 5I), as well as the ability of migration and tube formation in HUVECs (Fig. 5J–M). IHC analysis results showed that the expression of vWF was decreased in sh-circEGFR tumors compared with the control tumors (Fig. 2P). ELISA results showed that circEGFR reduced the levels of PLG (Fig. S3F). Next, we explored whether circEGFR promoted the conversion of PLG to plasmin through ANXA2 on the PM. Using the competitive ANXA2 inhibitor LCKLSL (an N-terminal hexapeptide that inhibits the binding of tissue plasminogen activator (tPA) to ANXA2 on the PM), ELISA results showed that LCKLSL treatment elevated the levels of PLG and partially rescued the inhibition of circEGFR overexpression on PLG levels (Fig. S3G and S3H). Moreover, LCKLSL rescued the ability of migration and tube formation of HUVECs promoted by circEGFR overexpression (Fig. S3I–L). These data elucidate that the translocation of ANXA2 toward the PM induced by circEGFR promotes angiogenesis in TNBC cells (Fig. 5N).

### CircEGFR plays a role as a sponge for autophagy-related miR-224-5p

**Fig. 6:**
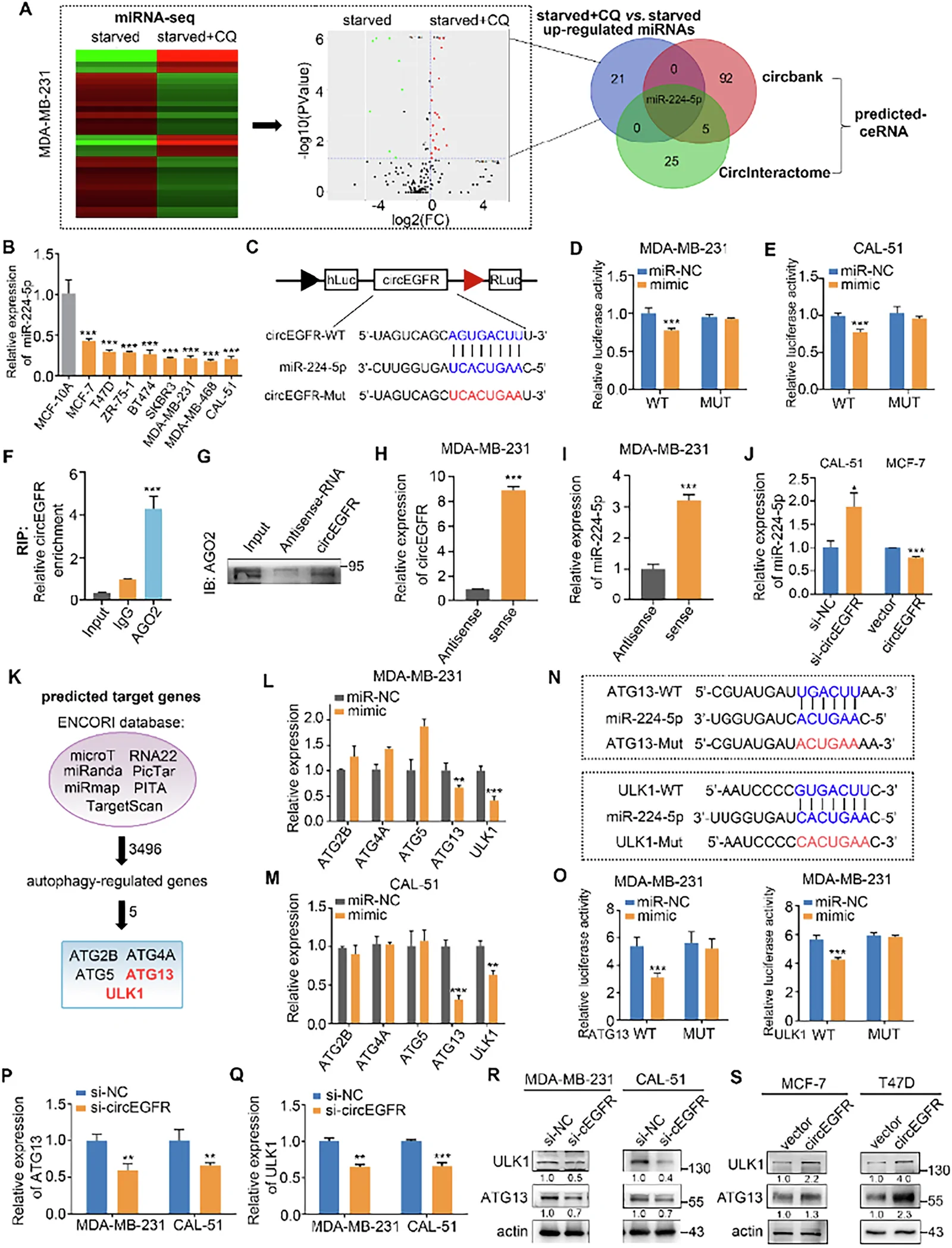
CircEGFR plays a role as a sponge for autophagy-related miR-224-5p. **A** Venn diagram of upregulated miRNAs in CQ-treated starved cells versus starved cells from RNA sequencing and predicted binding miRNAs showed circEGFR might function as a sponge for miR-224-5p. **B** The expression of circEGFR in MCF10A cells and breast cancer cells. **C** A diagram of circEGFR-WT and MUT luciferase reporter vectors. Luciferase activity was detected in (**D**) MDA-MB-231 and (**E**) CAL-51 cells co-transfected of circEGFR-WT plasmid or circEGFR-MUT plasmid and miR-224-5p mimics or miR-NC. **F** RIP assays was conducted in MDA-MB-231 cells using AGO2 antibody. The enrichment of circEGFR was determined by qPCR analysis. RNA pulldown was performed in MDA-MB-231 cells using Biotin-labeled circEGFR probes. The enrichment of (**G**) circEGFR and (**H**) miR-224-5p was determined by qPCR analysis. The enrichment of (**I**) AGO2 was determined by Western Blot analysis. **J** The expression of miR-224-5p in CAL-51 and MCF-7 cells transfected with si-circEGFR or circEGFR plasmid. **K** Venn diagram of the predicted target genes of miR-224-5p. The expression of 5 predicted target genes in (**L**) MDA-MB-231 and (**M**) CAL-51 cells transfected with miR-224-5p mimics or miR-NC. **N** The schematic diagram of binding sites between miR-224-5p and ATG13/ULK1 3′UTR or ATG13/ULK1 3′UTR MUT. **O** Luciferase activity was detected in MDA-MB-231 cells co-transfected of ATG13/ULK1-WT plasmid or ATG13/ULK1-MUT plasmid and miR-224-5p mimics or miR-NC. The expression of (**P**) ATG13 and (**Q**) ULK1 in MDA-MB-231 and CAL-51 cells transfected with si-circEGFR or si-NC. Western Blot results of (**R**) ULK1 and **S** ATG13 in MDA-MB-231 and CAL-51 cells transfected with si-circEGFR or si-NC. Data are shown as the means ± SD. *, *P* < 0.05; **, *P* < 0.01; ***, *P* < 0.001.

Considering that circRNA could play the role of ceRNA in tumors, we aimed to seek autophagy-associated miRNAs bound to circEGFR. The miRNA sequencing of starved and CQ-treated starved MDA-MB-231 cells was performed, and 22 miRNAs were substantially increased in CQ-treated starved cells (GSA: HRA005938). Through overlapping between 22 miRNAs and miRNAs bound to circEGFR predicted in the database (circbank (http://www.circbank.cn/) and CircInteractome (https://circinteractome.irp.nia.nih.gov/)), we focused on only one miRNA, miR-224-5p (Fig. 6A). The qPCR results showed that miR-224-5p was significantly lower expressed in breast cancer cell lines than MCF10A (Fig. 6B). According to the binding site of circEGFR and miR-224-5p predicted by the database (Fig. 6C), the circEGFR-MUT plasmid was constructed and the dual luciferase reporter assay was performed. In MDA-MB-231 and CAL-51 cells, miR-224-5p overexpression markedly inhibited luciferase activity after co-transfection of circEGFR-WT plasmid and miR-224-5p mimics (Fig. 6D, E). However, after co-transfection of circEGFR-MUT plasmid and miR-224-5p mimics, overexpression of miR-224-5p could not significantly inhibit luciferase activity (Fig. 6D, E). The RIP results showed that circEGFR was significantly enriched in anti-AGO2 group compared with IgG group, indicating that circEGFR could bind to AGO2 (Fig. 6F). Additionally, RNA pulldown results also displayed that circEGFR sense probe could significantly bind to AGO2 compared with antisense probe (Fig. 6G), and the sense probe prominently enriched circEGFR and miR-224-5p, which further verified that circEGFR could markedly bind to miR-224-5p (Fig. 6H, I). The qPCR results revealed that circEGFR reduced the expression of miR-224-5p (Fig. 6J). These data indicate that circEGFR could act as a sponge of miR-224-5p.

### miR-224-5p inhibits autophagy and malignant phenotypes of breast cancer cells and is degraded during autophagic progression

Next, we continued to explore whether miR-224-5p is involved in the regulation of autophagy. The qPCR results showed that miR-224-5p was significantly reduced in the starved cells compared with CQ-treated starved MDA-MB-231 cells (Fig. S4A). In addition, studies have found that miR-224-5p is preferentially degraded in the process of autophagy in hepatocellular carcinoma [27]. During the period of autophagy (Fig. 1B), starved MDA-MB-231 cells were cultured with or without actinomycin D. The data showed that miR-224-5p expression was decreased in a time-dependent manner, and CQ treatment restored the reduction of miR-224-5p expression (Fig. S4B). It suggested that the stability of miR-224-5p was diminished in an autophagic microenvironment and miR-224-5p might play a role in inhibiting autophagy of breast cancer cells. We transfected mimics into MDA-MB-231 and CAL-51 cells for miR-224-5p overexpression, and transfected inhibitors into MCF-7 and T47D cells for miR-224-5p knockdown (Fig. S4C, D). miR-224-5p overexpression markedly inhibited autophagic flux of breast cancer cells (Fig. S4E, F). Moreover, Western Blot results showed that overexpression of miR-224-5p decreased the expression of LC3II and promoted the expression of p62 in breast cancer cells, while knockdown of miR-224-5p had the opposite effect (Fig. S4G, H). These results indicated that miR-224-5p could reduce the autophagy levels of breast cancer cells.

In view of the relationship between autophagy and malignant progression in breast cancer, we investigated the function of miR-224-5p on breast cancer progression. Overexpression of miR-224-5p inhibited proliferation (Fig. S5A) and colony formation (Fig. S5B, C) of breast cancer cells, and miR-224-5p knockdown exerted the opposite effect (Fig. S5A–C). miR-224-5p overexpression also promoted G1 phase arrest and apoptosis of breast cancer cells (Fig. S5D–G). TCGA database analysis results showed that miR-224-5p was significantly lower expressed in breast cancer tissues compared with normal breast tissues (Fig. S5H). Compared with paracancer tissues, the expression of miR-224-5p in breast cancer tissues was significantly lower (Fig. S5I). Kaplan‒Meier Plotter database (https://kmplot.com/analysis/) showed that low expression of miR-224-5p was associated with poor prognosis of breast cancer patients (Fig. S5J). Our results indicate that miR-224-5p plays the function of tumor suppressor gene and inhibits autophagy in breast cancer cells. In addition, miR-224-5p could be degraded under the influence of circEGFR-induced autophagy, thereby weakening its inhibition of autophagy and malignant phenotypes in breast cancer cells.

### ATG13 and ULK1 are target genes of miR-224-5p and are upregulated by circEGFR

In order to further investigate the mechanism of circEGFR as an miR-224-5p sponge involved in the regulation of autophagy, five candidate target genes related to autophagy were predicted and screened through ENCORI database (https://starbase.sysu.edu.cn/) (Fig. 6K). The qPCR results displayed that only the expression levels of ATG13 and ULK1 were significantly reduced by miR-224-5p overexpression among the five candidate target genes (Fig. 6L, M). The 3’ UTR mutant plasmid was constructed according to the predicted binding sites of miR-224-5p and ATG13 or ULK1 (Fig. 6N). The dual luciferase reporter assay results showed that overexpression of miR-224-5p reduced luciferase activity after co-transfection of ATG13 WT or ULK1-WT and miR-224-5p mimics. However, after co-transfection of ATG13 MUT or ULK1-MUT and miR-224-5p mimics, miR-224-5p overexpression did not reduce luciferase activity in MDA-MB-231 and CAL-51 cells (Fig. 6O, Fig. S6A and S6B). The qPCR results revealed that overexpression of miR-224-5p decreased the mRNA expression of ATG13 and ULK1, similar to the effect of si-ATG13 and si-ULK1 (Fig. S6C, D). Moreover, miR-224-5p overexpression also diminished the protein expression of ATG13 and ULK1 (Fig. S6E, F). These results indicated that ATG13 and ULK1 are the target genes of miR-224-5p.

To further verify whether circEGFR could function as a sponge of miR-224-5p, the qPCR and Western Blot were performed. The results showed that decreased circEGFR expression reduced mRNA and protein levels of ATG13 and ULK1 in MDA-MB-231 and CAL-51 cells (Fig. 6P–S). We found that circEGFR expression was positively correlated with the expression of ATG13 and ULK1 in xenograft tumors (Fig. 2M, N). IHC analysis results showed that the expression of ATG13 and ULK1 was decreased in sh-circEGFR tumors compared with the control tumors (Fig. 2P). Moreover, the results showed that MDA-MB-231-derived exosomes inhibited the expression of ATG13 and ULK1 in ZR-75-1 cells transfected with si-circEGFR (Fig. 3H). Kaplan‒Meier Plotter database showed that ATG13 was significantly higher expressed in breast cancer tissues compared with normal breast tissues (Fig. S7A), and UALCAN database (http://ualcan.path.uab.edu/) results revealed that ULK1 was significantly higher expressed in breast cancer tissues than normal breast tissues (Fig. S7B). The above data suggest that high expression of circEGFR acts as a sponge of miR-224-5p to reduce the targeted inhibition effect of miR-224-5p on ATG13 and ULK1, enhancing the autophagy levels, thereby facilitating malignant phenotypes of TNBC cells.

### CircEGFR promotes autophagy and malignant phenotypes of TNBC cells by regulating miR-224-5p/ATG13/ULK1 axis

**Fig. 7:**
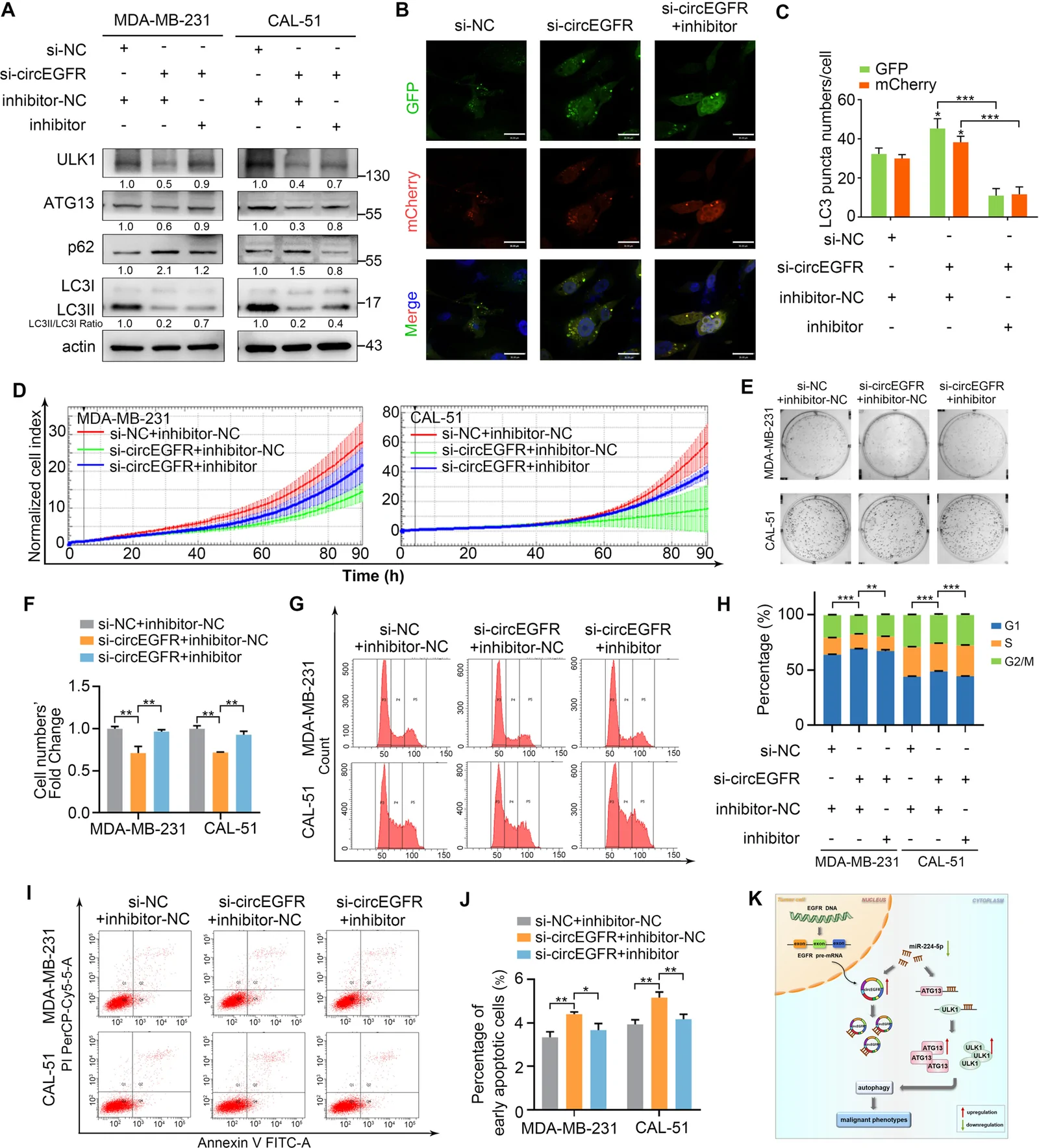
CircEGFR promotes autophagy and malignant phenotypes of TNBC cells by regulating miR-224-5p/ATG13/ULK1 axis. **A** Western Blot results of ULK1, ATG13, and autophagy-related protein in MDA-MB-231 and CAL-51 cells transfected with si-circEGFR or miR-224-5p inhibitors. **B** Representative images of autophagy levels in GFP-mCherry-LC3B-labeled MDA-MB-231 cells transfected with si-circEGFR or miR-224-5p inhibitors were observed by IF. Scale bar, 30 μm. **C** Quantitative analysis of LC3 puncta numbers in (**B**). **D** The xCELLigence RTCA-MP system was used to analyze cell proliferation after knocking down circEGFR or miR-224-5p in MDA-MB-231 and CAL-51 cells. **E** Colony formation assay was performed to analyze on cell colony formation after knocking down circEGFR or miR-224-5p in MDA-MB-231 and CAL-51 cells. **F** Quantitative analysis of cell numbers in (**E**). **G** Flow cytometry was used to analyze cell cycle in MDA-MB-231 and CAL-51 cells transfected with si-circEGFR or miR-224-5p inhibitors. **H** Quantitative analysis of cell cycle in (**G**). **I** Flow cytometry was performed to determine cell apoptosis after knocking down circEGFR or miR-224-5p in MDA-MB-231 and CAL-51 cells. **J** Quantitative analysis of cell apoptosis in (**I**). **K** The schematic diagram of circEGFR/miR-224-5p/ATG13/ULK1 axis in TNBC cells. Data are shown as the means ± SD. *, *P* < 0.05; **, *P* < 0.01; ***, *P* < 0.001.

**Fig. 8:**
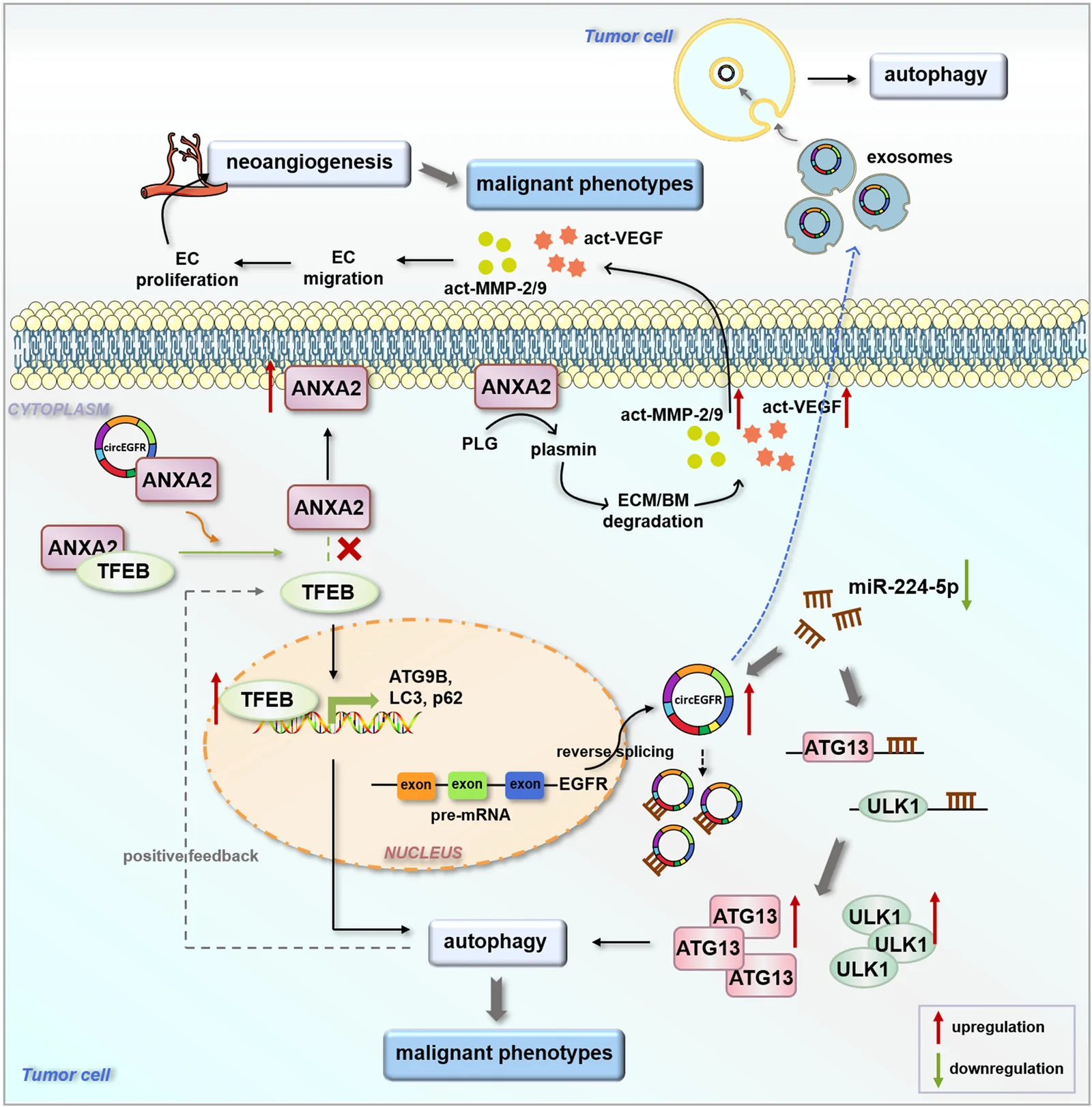
Schematic diagram of tumor-derived exosomal circEGFR facilitating TNBC autophagy. Tumor-derived exosomal circEGFR regulates TFEB nuclear trafficking by complexing with ANXA2 and miR-224-5p/ATG13/ULK1 axis to facilitate autophagy in TNBC.

To further verify the hypothesis, the rescue experiments of phenotypes were conducted. Western Blot results showed that co-transfected of si-circEGFR and miR-224-5p inhibitors partly reversed reduced ATG13 and ULK1 expression, while p62 expression was decreased, and LC3II expression was increased (Fig. 7A). In GFP-mCherry-LC3B-labeled MDA-MB-231 cells, we found that co-transfection of si-circEGFR and miR-224-5p inhibitors partly restored the decrease of autophagic flux (Fig. 7B, C). Then, we further determined whether circEGFR could interact with miR-224-5p to promote the malignant phenotypes of TNBC cells. We observed that the reduced proliferation and colony formation of MDA-MB-231 and CAL-51 cells was partially reversed by miR-224-5p inhibitors (Fig. 7D, E). miR-224-5p inhibitors partially restored G1 phase arrest and cell apoptosis induced by si-circEGFR (Fig. 7F–J). These data suggest that circEGFR enhances the autophagy levels and malignant phenotypes of TNBC cells by regulating miR-224-5p/ATG13/ULK1 axis (Fig. 7K). Our work highlights the mechanism underlying circEGFR regulating TFEB nucleus trafficking through complexing with ANXA2 and modulating miR-224-5p/ATG13/ULK1 axis to promote autophagy in TNBC (Fig. 8).

## Discussion

Autophagy increases tumor cell invasiveness and tolerance to stressful conditions during metastasis [28], which may be one of the reasons why TNBC is more aggressive and relapses earlier than other subtypes. There is growing evidence that autophagy facilitates proliferation, migration, self-renewal, and drug resistance of TNBC cells [29]. Studies have shown that autophagy-related proteins including ATG9 and LC3 are expressed in TNBC cells higher than in other subtypes [30, 31], and silencing of Beclin1, ATG5, and ATG7 leads to proliferation inhibition of TNBC cells [32]. Hence, autophagy is a promising target for TNBC. However, few studies have reported that circRNAs affect TNBC progression by regulating autophagy. In our study, we identified a circRNA that promotes autophagy in TNBC, circEGFR, which is upregulated in TNBC and correlated positively with lymph node metastasis tissues. Therefore, circEGFR is expected to be encouraging biomarker and target for TNBC.

Annexins are a family of calcium-regulated phospholipid-binding proteins. All annexins have an analogous core domain including four repetitive α-helical for Ca^2+^ binding and have a high affinity for membrane phospholipids. Each annexin has a distinctive N-terminal tail domain that leads to interaction with other proteins [26]. ANXA2 is a member of the annexin family. It exists as a monomer in the nucleus and as a heterotetrameric form in the cytoplasm. In breast cancer, analysis of plasma samples from patients shows that ANXA2 levels are significantly higher than normal plasma, suggesting that ANXA2 could act as a predictor for breast cancer development [33]. Furthermore, gene expression analysis of TNBC patients supports these observations and shows that high expression of ANXA2 is associated with decreased overall survival and recurrence-free survival [34]. ANXA2 has been reported to involve in autophagy induction. Several studies indicated that ANXA2 participates in autophagy after interferon-γ (IFN-γ) stimulation, which fuses with multivesicular bodies (MVBs) but not lysosomes and translocate toward the PM [35]. ANXA2 interacts with phosphorylated mTOR to change its cellular localization and induce autophagy fluctuations [15]. ANXA2 interacts with TRIM21 to participate in autophagy in osteosarcoma cells [20]. In our study, as an interaction partner of circEGFR, ANXA2 was essential for circEGFR-induced autophagy in breast cancer cells. Although circEGFR had little effect on the mRNA and protein expression of ANXA2, it promoted the translocation of ANXA2 to the PM in breast cancer cells. Mechanistically, we demonstrated that circEGFR separated the ANXA2-TFEB complex by combining with ANXA2, and facilitated ANXA2 plasma membrane trafficking and TFEB nucleus translocation. TFEB, as a transcription factor for autophagy-related proteins, including ATG9B, LC3, and p62, promotes autophagy process in tumors [23]. We observed that TFEB enters the nucleus in the cells exposed to starvation, suggesting that circEGFR-induced autophagy could form a positive feedback loop to promote higher autophagy levels. Studies have indicated that ANXA2 expressed on the cell surface is a significant mediator of signaling pathways in the tumor microenvironment (TME) [36] and is associated with poor differentiation, relapse, decreased patient survival, neovascularization, and immunosuppression [37–40]. Our study confirmed that knockdown of circEGFR in breast cells reduces ANXA2 expression on the PM, leading to a decrease in the expression of active MMP-9 and VEGFA, and the ability of migration and tube formation in HUVECs treated with conditioned medium derived from the cells stably circEGFR knockdown were significantly reduced. These results could serve as another powerful clue for circEGFR as a biomarker for diagnosing TNBC.

There is growing evidence that exosomes naturally exist in body fluids including plasma, and can carry a variety of important information such as proteins, lipids, and RNA, and participate in intercellular communication [41]. Some studies have found that circRNAs in exosomes are at least 2-fold enriched in their producer cancer cells, and the ratio of circRNAs to linear RNAs in exosomes is about 6-fold higher than in cells [5, 41]. Therefore, compared with tumor tissues, the stable presence of circRNAs in exosomes provides a more convenient method for tumor diagnosis and circRNAs are potential targets for cancer therapy. Tumor cells are surrounded by TME, which consists of tumor cells, tumor-infiltrating immune cells, lymphatic vessels, blood vessels, stromal cells, and cytokines [42]. Autophagy has different effects on tumor progression in different microenvironmental cells. By selectively inhibiting ULK1 in leukemia stem cells, oxidative stress-induced differentiation is ameliorated, resulting in increased sensitivity to chemotherapy [43]. A cell-permeable derivative of α-KG (DMKG) significantly reduces autophagy in tumor cells which in turn leads to increased release of antigens and inflammatory factors, thereby activating DC cells and improving anti-tumor immunity [44]. TGF-β1-activated CAFs promote breast tumor invasion and metastasis via the autophagy process [45]. Moreover, malignant tumor induces autophagy in the microenvironment and distal tissues. Previous studies showed that autophagy in normal epithelial cells induced by tumor cells provides recyclable nutrients including amino acids to support tumor cell proliferation. Autophagy-deficient tumors are dormant but resume growth in an autophagy-rich microenvironment [46]. In our study, circEGFR was highly expressed in exosomes from the plasma of breast cancer patients. TNBC cells formed a circEGFR-riched microenvironment and increased autophagy levels in other breast cancer cells, thereby accelerating breast cancer progression.

Several studies indicate that miR-224-5p plays a role in regulating autophagy in pancreatic mucinous cystadenocarcinoma and hepatocellular carcinoma, etc. [47, 48]. However, there is currently controversy over the role of miR-224-5p in the regulation of autophagy in breast cancer. Studies have showed that miR-224-5p carried by human umbilical cord mesenchymal stem cells-derived exosomes promotes the autophagy and proliferation of breast cancer cells [49]. miR-224-5p suppresses autophagy levels of breast cancer cells by targeting Smad4 [50]. In our study, we found that miR-224-5p was downregulated in breast cancer cells and tissues, and inhibited the autophagy process of breast cancer cells. Studies have indicated that miR-224-5p could be preferentially degraded under autophagy in hepatocellular carcinoma [27], and we found that miR-224-5p was also preferentially degraded under autophagic conditions in breast cancer cells, allowing less miR-224-5p to exert an autophagy-suppressing effect. Our results showed that circEGFR reduced the expression of miR-224-5p, and circEGFR-induced autophagy might be involved in the degradation of miR-224-5p, thereby reducing its inhibition of autophagy, which forms a positive feedback loop for autophagy. Our findings provide a new understanding of the regulatory mechanism of the autophagy process mediated by circEGFR to accelerate TNBC progression, suggesting exosomal circEGFR as a promising biomarker and target for TNBC therapy.

## Methods

### Clinical samples

Human breast cancer tissues (85 pairs of tumor and adjacent tissues) of Her2, Luminal and TNBC were obtained from Cancer Hospital, the Chinese Academy of Medical Sciences, and Peking Union Medical College. The plasma samples of 72 breast cancer patients and 24 healthy people were collected from Cancer Hospital, the Chinese Academy of Medical Sciences, and Peking Union Medical College. The tissues and plasma have obtained informed consent from all patients. The clinical information of the tissues and plasma was shown in Table S1 and S2. Our work was approved by the Ethics Committee of Cancer Hospital, Chinese Academy of Medical Sciences/ National Cancer Center (No. NCC2018–043).

### RNA extraction and qPCR

The collected cells, the frozen tissues and plasma-derived exosomes were added with Trizol (Invitrogen, USA), and RNA was extracted by RNAExpress Total RNA Kit (NCM Biotech, China). Reverse transcription was performed by Superscript II reverse transcriptase (Invitrogen, USA). RT-qPCR was conducted by BlastaqTM 2X qPCR MasterMix (ABM, Jiangsu, China) using CFX96 Touch Real Time PCR Detection System (Bio Rad, Hercules, California, USA). The internal reference of circRNAs and mRNAs is GAPDH, the internal reference of miRNAs is U6, and the external reference of circEGFR in plasma-derived exosomes is a long-chain noncoding RNA GM13008, which is expressed only in mice and not in humans. The 2^−△△Ct^ is used to analyze the results, and Ct value represents the threshold cycle. The sequences of qPCR Primers in our work were shown in Table S3.

### Lentiviral infection

A luciferase-labeled lentivirus stably knocking down circEGFR (sh-circEGFR) and its negative control (sh-NC) were constructed by Genechem (Shanghai). CAL-51 cells were infected by the lentiviruses with a multiplicity of infection of 100 and then were selected with puromycin (2 μg/mL, Solarbio, China). The knockdown efficiency of circEGFR was detected by qPCR.

### Flow cytometry analysis

#### Cell cycle analysis

The cells were starved overnight to synchronize. The cells were fixed overnight with 70% pre-cooled ethanol, washed with phosphate buffered saline (PBS), stained with 300 μL PI (BD Biosciences, NJ, USA), and incubated at room temperature for 15 min. Flow cytometry (BD Biosciences) was used to detect the proportion of cell cycle.

#### Cell apoptosis analysis

After cell transfection, the supernatant and cells were collected through centrifugation. The precipitates were suspended in 600 μL binding buffer and added with 5 μL Annexin V-FITC (KeyGene Biotech, China). After mixing, 5 μL propidium Iodide (PI) (KeyGene Biotech, China) was added, and the reaction was conducted at room temperature in a dark environment for 15 min. Flow cytometry was used to detect the percentage of cell apoptosis.

### Angiogenesis

#### Tube formation assay

The conditioned medium of sh-circEGFR and sh-NC cells was collected. 50% matrigel was added to 96 well plate. HUVEC cells (2 × 10^4^/well) were suspended in 100 μL conditioned medium and were seeded on matrigel. Tube formation of HUVEC cells was observed at 37 °C for 3 h, and branch numbers and capillary length of each well were counted by Image J.

#### Migration assay of HUVEC cells

the conditioned medium of sh-circEGFR and sh-NC cells collected was added to the lower chamber of Transwell (Corning, NY, USA), and 100 μL DMEM was used to suspend HUVEC cells in the upper chamber. The migration of HUVEC cells was observed at 37 °C for 3 h. Image J was used for the cell count, and GraphPad Prism was used for the above result statistics.

### Immunofluorescence (IF)

MDA-MB-231 cells were infected by an adenovirus expressing mCherry-GFP-LC3B fusion protein. During the fusion of autophagosome and lysosome, GFP fluorescence is quenched in the acid environment of the lysosome, but mCherry fluorescence is retained because of its stability, which can be used for the detection of autophagic flux of cells. mCherry-GFP-LC3B-labeled MDA-MB-231 cells were seeded into the chamber to observe the changes of autophagic flux.

In order to observe the effect of circEGFR on the localization of ANXA2 and TFEB in breast cancer cells, the primary antibodies ANXA2 and TFEB were incubated, and the corresponding secondary antibodies were FITC-labeled goat anti-Mouse IgG (H + L) and Alexa Fluor® 594-labeled goat anti-Rabbit IgG (H + L) respectively, and DAPI. The confocal laser scanning microscopy (Olympus, Miyazaki, Japan) was used for observation. A full list of antibodies used in this study is provided in Table S4.

### Western blot

Analysis of protein expression by SDS-PAGE was performed, using the primary antibodies including anti-p62, anti-LC3, anti-GAPDH, anti-ULK1, anti-ATG13, anti-EGFR, anti-ANXA2, anti-TFEB, anti-Lamin B, anti-MMP-9, anti-VEGF, anti-HSP70, anti-Alix, and anti-CD63, and secondary antibody including anti-Rabbit IgG (H + L) and anti-Mouse IgG (H + L), the proteins were visualized using chemiluminescence (ImageQuant LAS 4000, General Electric Company, USA). A full list of antibodies used in this study is provided in Table S4.

### Exosome experiments

#### Extraction of plasma-derived exosomes

The residual cells were removed by 2000 *g* centrifugation for 20 min, 10000 g centrifugation for 20 min, and 1/3 volume of Ribo™ Exosomes Isolation Reagen (RiboBio, China) was added, standing at 4 °C for 30 min, centrifuging 15,000 *g* for 2 min, and aspirating the supernatant.

#### Collection and identification of cell-derived exosomes

MDA-MB-231 cells were cultured in the DMEM without exosomes. The conditioned medium was collected, and then centrifugated at 1400 rpm for 20 min after 48 h. The supernatant was centrifuged at 12000 rpm for 1 h, and at 150,000 g for 70 min twice to obtain the precipitation. The size and morphology of the precipitate were observed by transmission electron microscope (TEM) (HITACHI, Japan) and the quality was evaluated by Nanosight (Malvern, UK). The precipitate was added with loading buffer for Western Blot detection of exosome marker including HSP70, Alix, and CD63.

#### Exosomes experiment in vitro

The cells (8 × 10^5^/well) to be treated were seeded into a 6-well plate, and then the exosomes from MDA-MB-231 were added and co-cultured for 36 h. The cells treated were collected for subsequent experiments.

### Animal studies

The 4-week-old female NOD/SCID mice were purchased from Charles River (Beijing, China), and sh-circEGFR and sh-NC cells (5 × 10^6^) were subcutaneously implanted to the mammary fat pads on both sides of each NOD/SCID mice (*n* = 10 per group). The tumor volume was monitored weekly using the formula (L × W^2^)/2 (L is the length and W is the width of the tumor). Ten weeks later, the mice were euthanized, and the tumor weight was measured. The 4-week-old female BALB/C nude mice were purchased from Charles River, and sh-circEGFR and sh-NC cells (1 × 10^6^) were injected into the tail vein of mice (*n* = 10 per group). Eight weeks later, the lungs were screened by optical in vivo Imaging System (PE, USA). The mice were euthanized, and the lungs were collected and analyzed by H&E. Animal experiments in our study were approved by the Institutional Animal Care and Use Committee of National Cancer Center/National Clinical Research Center for Cancer/Cancer Hospital, the Chinese Academy of Medical Sciences, and Peking Union Medical College (No. NCC2018A014).

### Statistical analysis

In our study, GraphPad Prism 8 and Image J were used for statistics of data and images. The data displayed in this study were mean ± SD. The two-tailed Student’s *t* test was used for difference analysis between any two groups, one-way analysis of variance followed by Bonferroni post *hoc* test was applied for difference analysis among multiple groups. The spearman correlation was used to calculate r and *P*-values. For clinical data, the chi-square test and Fisher’s exact test were used for classification variables. The statistical significance was defined as *P < 0.05, **P < 0.01, and ***P < 0.001. In vitro experiments were repeated for three times if not specifically indicated.

## Supplementary information

Supplementary information

## Data Availability

The datasets used and analyzed during the current study are available from the corresponding author on reasonable request. The raw transcriptome data have been deposited to the Genome Sequence Archive. GSA accession numbers: HRA005935; HRA005938.
