# Correction: Novel exosomal circEGFR facilitates triple negative breast cancer autophagy via promoting TFEB nuclear trafficking and modulating miR-224-5p/ATG13/ULK1 feedback loop

**DOI:** 10.1038/s41388-026-04010-5

**Published:** 2026-10-08

**Authors:** Huachen Song, Zitong Zhao, Liying Ma, Weihong Zhao, Yi Hu, Yongmei Song

**Affiliations:** 1 Senior Department of Oncology, the Fifth Medical Center, Chinese PLA General Hospital, Beijing, 100853 China; 2 State Key Laboratory of Molecular Oncology, National Cancer Center/National Clinical Research Center for Cancer/Cancer Hospital, Chinese Academy of Medical Sciences and Peking Union Medical College, Beijing, 100021 China; 3 Department of Medical Oncology, Chinese PLA General Hospital, Beijing, 100853 China

Correction to: *Oncogene* https://doi.org/10.1038/s41388-024-02950-4, published online 27 January 2024

Following the publication of this article, the authors noted an error in Figure 6S. Specifically, during figure assembly an incorrect loading control image for the T47D cell line was included. Figure 6S has now been updated and is included below.

The authors have verified that the error is strictly confined to the figure presentation and does not affect the interpretations, or conclusions in the paper. The authors apologize for this oversight and any resulting inconvenience to the readers.

Former Fig 6:

**Figure:**
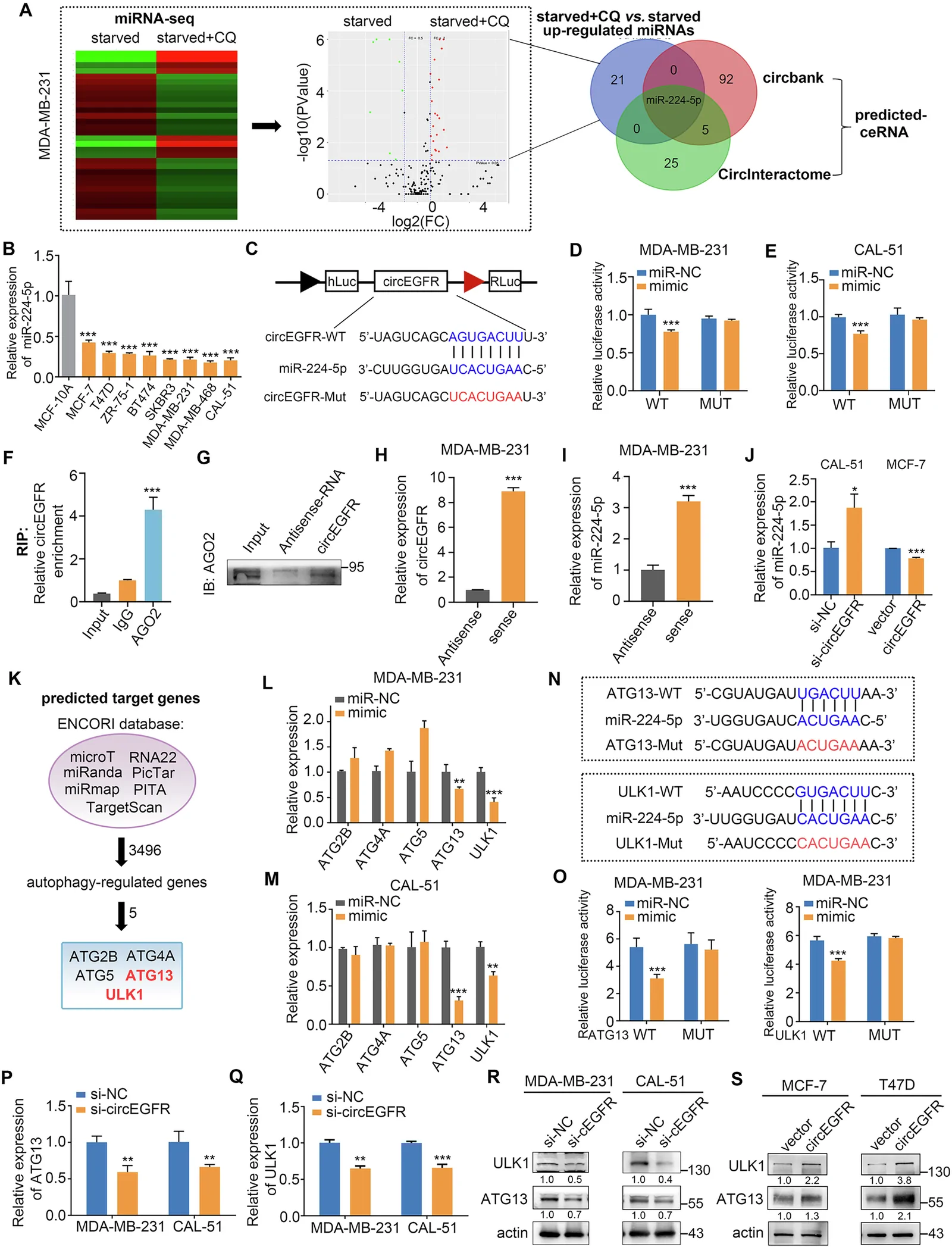

Amended Fig 6:

**Figure:**
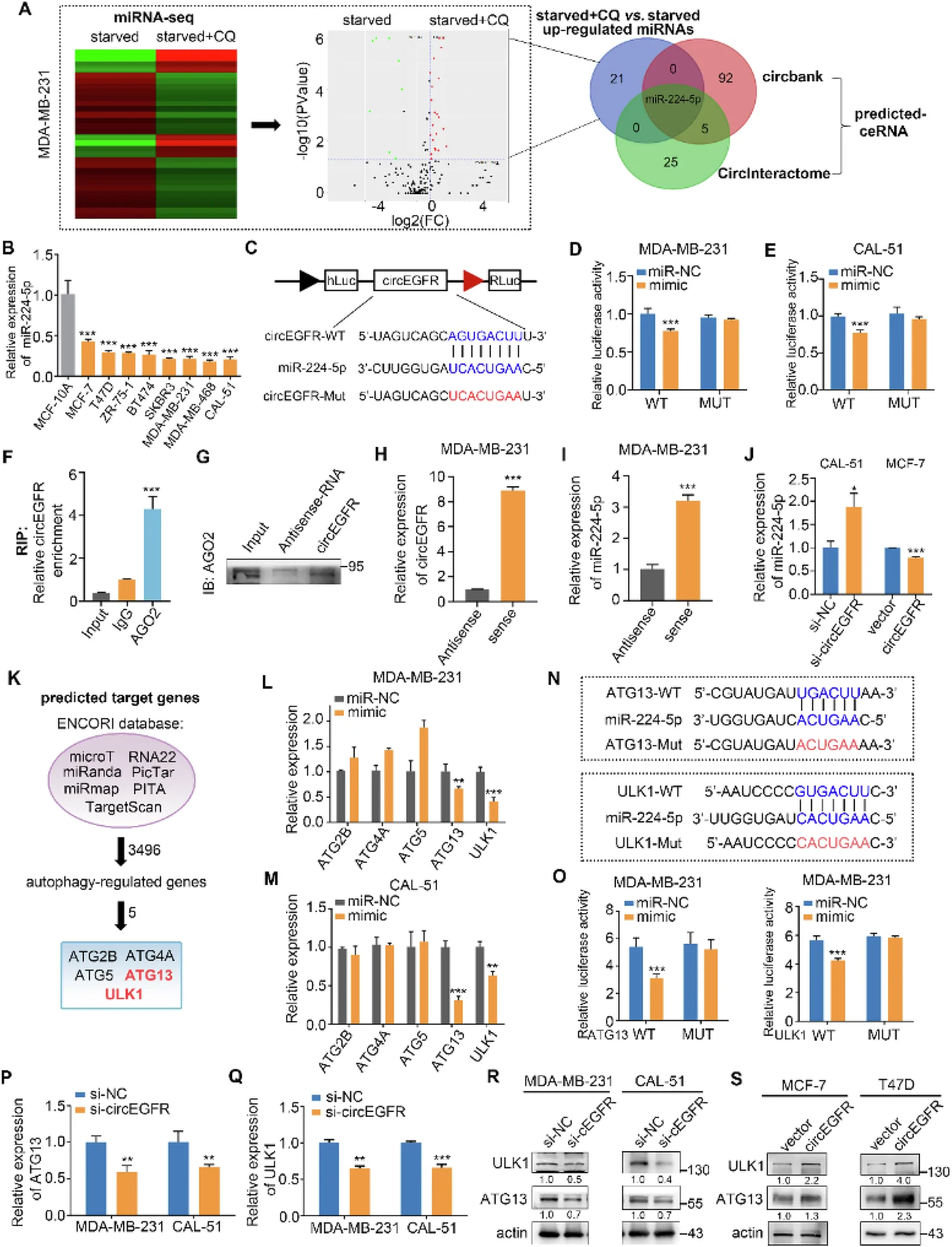

The original article has been corrected.

