# Supplementary material for: Novel exosomal circEGFR facilitates triple negative breast cancer autophagy via promoting TFEB nuclear trafficking and modulating miR-224-5p/ATG13/ULK1 feedback loop
Source: Oncogene. 2024 Jan 27;43(11):821–36. doi: 10.1038/s41388-024-02950-4 (PMC10920198; doi:10.1038/s41388-024-02950-4)
Supplement: Supplementary file 1 — Supplementary information [file 41388_2024_2950_MOESM1_ESM.doc]

**Supplementary materials and methods**

**Cell culture and transfection**

The human breast cancer cell lines HCC1937, MDA-MB-231, MDA-MB-468, CAL-51, T47D, BT474, MCF-7, and ZR-75-1 (gifted by Prof. Chen Lin, Cancer Hospital, the Chinese Academy of Medical Sciences, and Peking Union Medical College) were cultured in DMEM (Gibco, USA) supplemented with 10% fetal bovine serum (FBS). The human mammary epithelial cell MCF10A (gifted by Prof. Zhihua Liu, Cancer Hospital, the Chinese Academy of Medical Sciences, and Peking Union Medical College) was cultivated in DMEM/F12 (Gibco) supplemented with 5% horse serum, insulin (10 μg/mL), EGF (20 ng/mL), choleragen (100 ng/mL), and hydrocortisone (0.5 μg/mL). Human umbilical vein endothelial cells (HUVECs) (conserved in State Key Laboratory of Molecular Oncology, Cancer Hospital, the Chinese Academy of Medical Sciences, and Peking Union Medical College) were cultured in DMEM supplemented with 10% FBS. The cells were cultured in an incubator at 37°C containing 5% CO_2_. All cell lines used were confirmed the authentication. Small interfering RNA (siRNA) of circEGFR was purchased from JTSBIO Co., Ltd. (Wuhan, China). siRNAs of ATG13 and ULK1 were purchased from JTSBIO Co., Ltd. miR-224-5p mimics, miR-224-5p inhibitors and corresponding negative controls (miR-NC or miR-NC inhibitor) were purchased from RiboBio (Guangzhou, China). The cells were transfected with siRNA or miRNA by Lipofectamine 2000 (Invitrogen, Carlsbad, CA, USA). The siRNA and miRNA sequences were listed in **Table S3**. The plasmid overexpressing circEGFR, ATG13, and ULK1 were constructed by GeneRay Inc. (Shanghai, China). The cells were transfected with the plasmids by Neofect^TM^ DNA transfection reagent (NEOFECT, China).

**Ribonuclease R (RNase R) digestion and actinomycin D assays**

To detect the resistance of circRNA to RNase R digestion, RNase R (1 U/µg RNA) was added to the extracted RNA. Reverse transcription was performed, and then the results were detected by qPCR.

In order to compare the stability of circRNA and linear RNA, actinomycin D (Sigma-Aldrich, MO, USA) was added to MDA-MB-231 and MCF-7 at 3 µg/mL and 6 µg/mL respectively. The samples were collected at 0, 4, 8, 12 h for qPCR detection. In order to prove that autophagy reduces the stability of miR-224-5p, actinomycin D was added to MDA-MB-231 at 3 µg/mL. The samples were collected at 0, 12, 24, 36, and 48 h for qPCR detection.

**Subcellular fraction assays**

The assay was performed according to the manufacturer’s protocol (Beyotime Biotechnology, China), and was used to analyze the expression of different components in cells. Briefly, cells were harvested and washed with ice-cold PBS for the preparation of nuclear and cytoplasm separation. Afterward, the cell precipitation was lysed by Cytoplasmic Protein Extraction Reagent A and Nucleus Protein Extraction Reagent B, respectively. The internal reference of cytoplasmic protein is GAPDH, and the internal reference of nuclear protein is Lamin B. Then, Western Blot and qPCR were used to analyze expression in different fractionations.

**Fluorescence in situ hybridization (FISH)**

The probe designed for circEGFR was used for in situ hybridization, and the assay was performed according to the manufacturer’s protocol (RiboBio, China). The cells were fixed at room temperature with 4% paraformaldehyde, then 1 mL of pre-cooled PBS containing 0.5% tritonX-100 (Solarbio, China) was added. The pre-hybridization solution was used to block at 37 ℃ for 30 min. 2 µL 100 µM circEGFR probe was added to 60 µL hybridization solution at 37 ℃ overnight. The cells were washed with SSC buffer of different concentrations and were incubated with fluorescent-labeled secondary antibody and DAPI at 37 ℃ for 1h. Finally, the results were photographed by confocal laser scanning microscopy. The sequences of FISH probes in our work were shown in **Table S3**.

**RNA immunoprecipitation (RIP)**

The cells were collected and washed with ice-cold PBS. The cells were added IP lysis buffer at 4 x 10^7^ cells/mL and incubated at 4 ℃ for 10 min. Then 25 µL of protein A/G beads (Santa Cruz, CA, USA) were suspended in 100 µL of binding buffer and added 2 µg of antibody (ANXA2 (Proteintech, 60051-1-Ig) or Ago2 (Proteintech, 10686-1-AP)) or corresponding IgG, incubated at room temperature for 1 h. After washing the beads, the samples were added 200 µL of cell lysate and 800 µL of binding buffer, and incubated for 6 h. The beads were washed 6 times, and then WB and qPCR assays were performed.

**The reporter construction and luciferase reporter assay**

Wild-type (WT) or mutant (MUT) 3′-UTR of circEGFR, ATG13, or ULK1 were cloned into the firefly-tagged pmirGLO promoter luciferase vector (GeneRay, China). MDA-MB-231 and CAL-51 were seeded in 48-well plates, and then transfected with mimic-NC or miR-224-5p mimic, circEGFR-WT or circEGFR-MUT, ATG13-WT or ATG13-MUT, and ULK1-WT or ULK1-MUT, respectively. After 48 h, the samples were processed according to the instructions of the Dual Luciferase Reporting and Detection System (Promega, W, USA) and detected with microarray microplate analyzer (PerkinElmer, MA, USA).

**Immunohistochemistry (IHC)**

Briefly, paraffin-embedded tumor tissue sections were deparaffinized with xylene and rehydrated in a gradient concentration of alcohol. The slides were processed for antigen recovery in Tris-EDTA buffer (10 mM, pH 8.0) at 100 °C for 15 min and then cooled naturally. Endogenous peroxidase activity was blocked by treatment with 3% hydrogen peroxide (H_2_O_2_) for 10 min. After blocking with 1% BSA solution for 30 min, the slides were incubated with primary antibodies (Ki67, ATG13, ULK1, ANXA2, TFEB, and vWF) respectively at 4°C overnight. The results were observed by an Aperio pathology slide scanner (Leica, Germany). A full list of primary antibodies used in this study is provided in Supplementary **Table S4**. The slides were then detected by using DAB, counterstained with hematoxylin, dehydrated, and mounted.

**Cell proliferation assay**

The transfected cells were seeded into E-Plate 96-well plates at 3 x 10^3^ cells/well. After 30 minutes of standing at room temperature, the E-Plate 96-well plates were placed into the xCELLigence Real-Time Cell Analyzer (RTCA)-MP system (Acea Biosciences/Roche Applied Science, CA, USA), and the system automatically recorded the cell index every 15 minutes.

**Transwell assay**

The migration assay: The transfected cells were resuspended at 2 x 10^5^ cells in 150 µL of serum-free medium, and added to the upper chamber. Then 600 µL of 20% FBS was added to the lower chamber.

The invasion assay: The procedure is similar to the migration assay, but a 2% matrigel solution should be prepared in serum-free medium in advance and added to the upper chamber.

After 15 hours of incubation, the upper chamber was taken out and fixed with methanol for 5 min, followed by 2% crystal violet solution at room temperature for 8 min. We performed cell counting on the images by Image J.

**Colony formation assay**

The transfected cells were seeded into 6-well plates at 1 x 10^3^ cells/well. The cells were cultured for about 7-10 days and washed with PBS for 2 times. The cells were fixed with methanol for 5 min and then stained with 2% crystal violet solution for 8 min. We performed cell counting on the images by Gel Imaging Analysis System (SYNGENE, Cambridge, UK).

**RNA pulldown and mass spectrometry analysis**

Biotinylated circEGFR probes were obtained from GeneRay. 5 µg NC probes and circEGFR probes and 25 µL Streptavidin beads were incubated at room temperature for 1h. The washed beads were added with 250 µL cell lysate and 800 µL binding buffer, incubating at room temperature for 1h. Then beads were washed for 8 times. The enriched miRNAs were detected by qPCR, and the enriched proteins were detected by Western Blot. The proteins binding with NC probes or circEGFR probes were visualized through silver staining. The thicker bands in circEGFR probes compared with NC probes were cut to perform mass spectrometry analysis by Novogene Co., Ltd. (Beijing, China). The sequences of NC probes and circEGFR probes were listed in **Table S3**.

**Co-immunoprecipitation (co-IP)**

The cells were lysed to obtain the cell lysate, and the concentration was measured. 2 mg of protein lysate was added with 2 µg of antibodies including ANXA2, TFEB, anti-Mouse IgG, and anti-Rabbit IgG, and incubated overnight at 4 ℃. 80 µL protein A/G beads were added to the mixture, and incubated for 4 h at 4 ℃. The beads were washed 8 times and centrifuged. The results were detected by Western Blot.

**Enzyme-linked immunosorbent assay (ELISA)**

The levels of PLG in the conditioned medium from MDA-MB-231 cells expressing circEGFR or vector and treated with or without LCKLSL (10 μM, MCE, NJ, USA) were measured using ELISA kits according to the manufacturer’s protocol (Cloud-Clone, China). Briefly, The standards and samples were added to the plate at 100 µL/well and incubated at 37°C for 1 h. The Detection Solution A was added at 100 µL/well and incubated at 37°C for 1 h. Then the plate was washed 3 times. The Detection Solution B was added at 100 µL/well and incubated at 37°C for 30 minutes. The plate was washed 5 times. TMB substrate was added 90 µL and incubated at 37°C for 10-20 min. Finally, 50 µL of termination solution was added and the results were immediately detected by microarray microplate analyzer at 450 nm.

**Supplementary Figure**


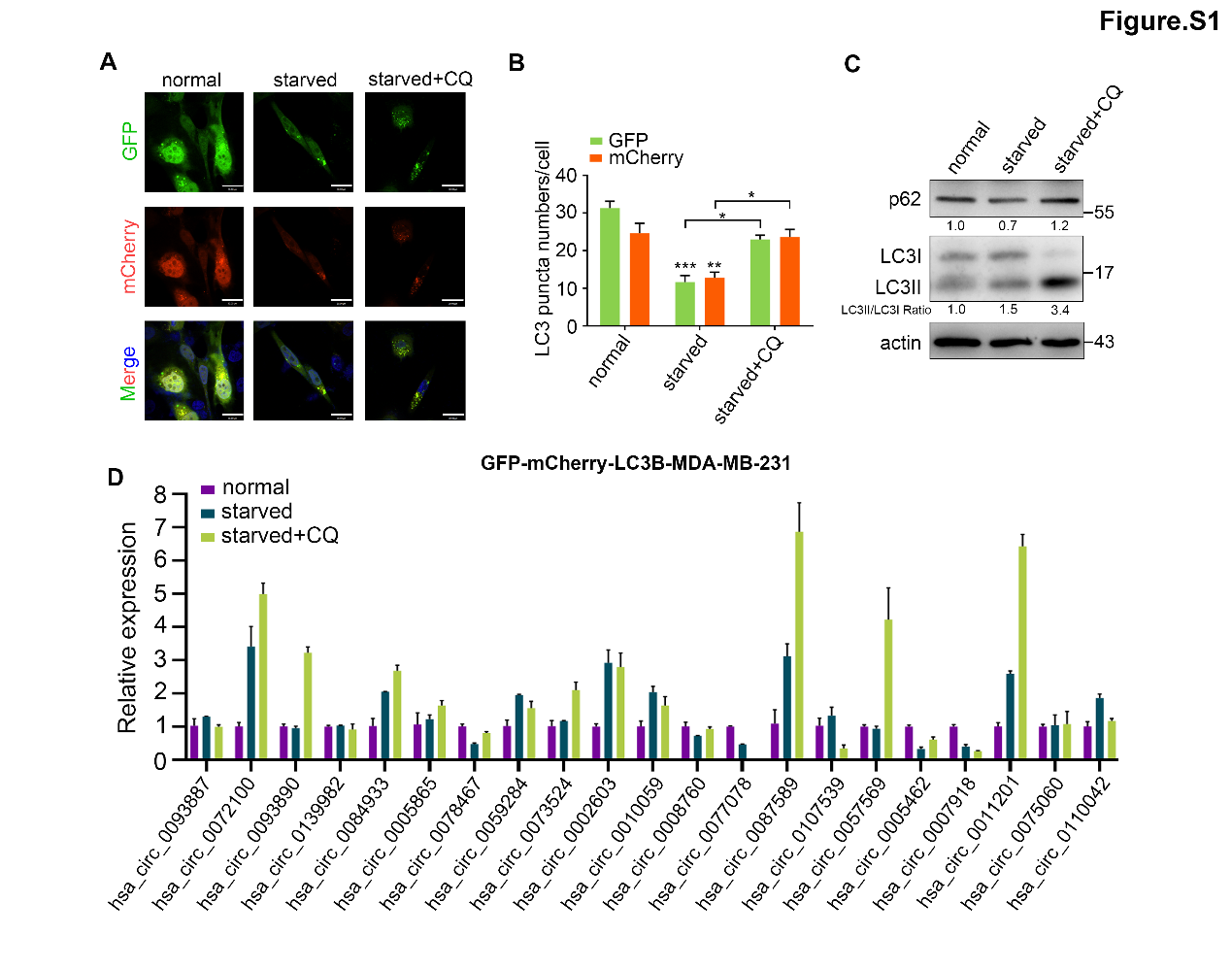


**Fig. S1 Amino acid deficiency promotes autophagy in TNBC cells.** **(A)** Representative images of autophagy levels in normal, starved (cultured with amino acid-free DMEM for 48 h), and CQ-treated starved GFP-mCherry-LC3B-labeled MDA-MB-231 cells (cultured with amino acid-free DMEM for 24 h and then treated with CQ for 24 h) were observed by IF. Scale bar, 30 μm. **(B)** Quantitative analysis of LC3 puncta numbers in Figure A. **(C)** Western Blot results of p62 and LC3II in normal, starved, and CQ-treated starved GFP-mCherry-LC3B-labeled MDA-MB-231 cells. **(D)** The expression of 21 circRNA candidates in normal, starved, and CQ-treated starved GFP-mCherry-LC3B-labeled MDA-MB-231 cells was determined by qPCR. Data are shown with the means ± SD. *, *P* < 0.05; **, *P* < 0.01; ***, *P* < 0.001.


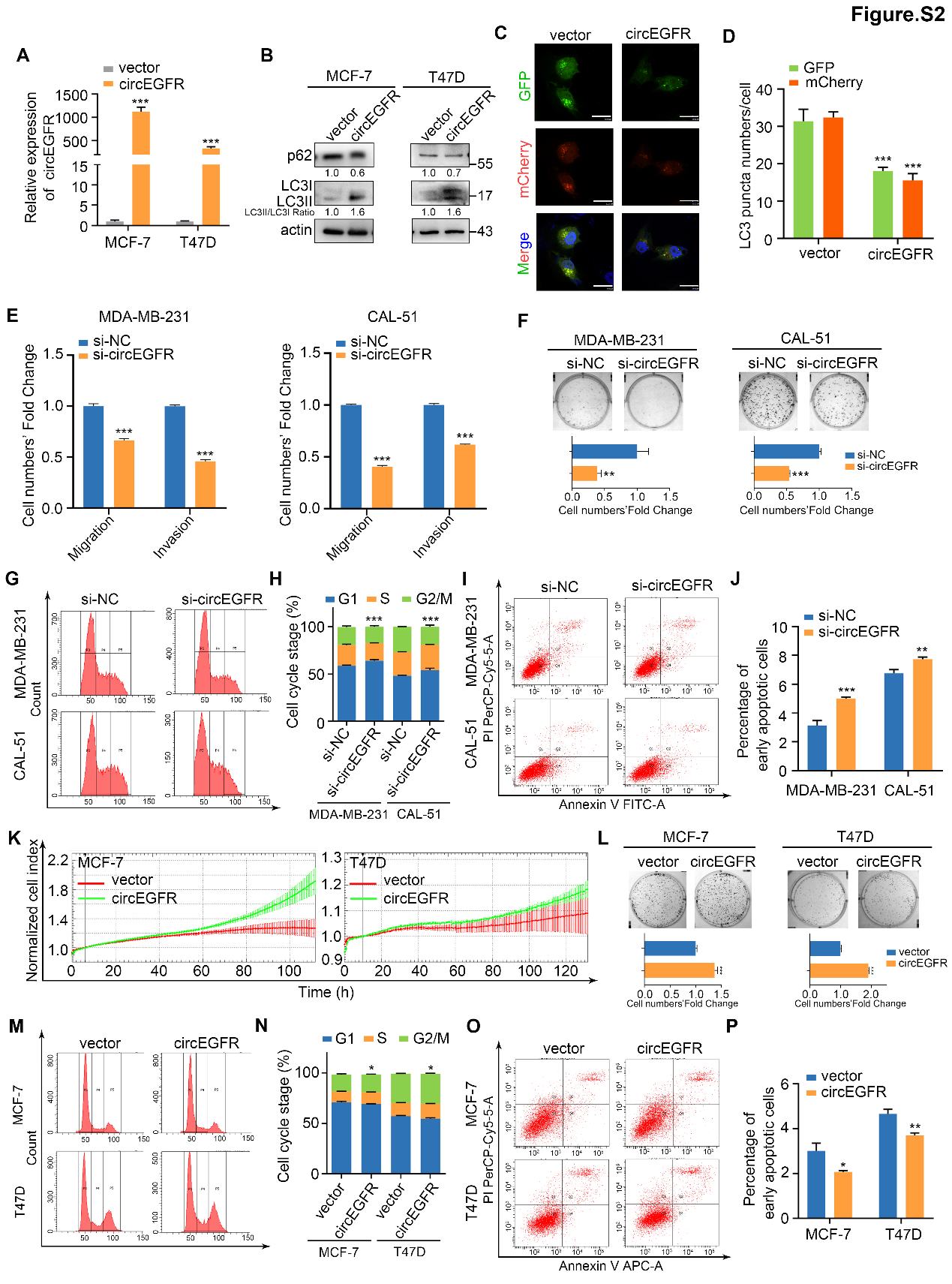


**Fig. S2** **circEGFR promotes autophagy levels and malignant phenotypes of TNBC cells *in vitro*. (A)** qPCR showed the expression of circEGFR in MCF-7 and T47D cells transfected with circEGFR or vector. **(B)** Western blot results of p62 and LC3II in MCF-7 and T47D cells transfected with circEGFR or vector. **(C)** Representative images of autophagy levels in MCF-7 and T47D cells after overexpressing circEGFR were observed by IF. Scale bar, 30 μm. **(D)** Quantitative analysis of LC3 puncta numbers in Figure C. **(E)** Quantitative analysis of cell migration and invasion in Figure 2F. **(F)** Colony formation analysis in MDA-MB-231 and CAL-51 cells. **(G)** Flow cytometry was used to analyze cell cycle in MDA-MB-231 and CAL-51 cells transfected with si-circEGFR or si-NC. **(H)** Quantitative analysis of cell cycle in Figure G. **(I)** Flow cytometry was performed to determine cell apoptosis after knocking down circEGFR in MDA-MB-231 and CAL-51 cells. **(J)** Quantitative analysis of cell apoptosis in Figure I. **(K)** The xCELLigence RTCA-MP system was used to analyze cell proliferation after overexpressing circEGFR in MCF-7 and T47D cells. **(L)** Colony formation assay was performed to analyze on cell colony formation after overexpressing circEGFR in MCF-7 and T47D cells. **(M)** Flow cytometry was used to analyze cell cycle in MCF-7 and T47D cells transfected with circEGFR or vector. **(N)** Quantitative analysis of cell cycle in Figure M. **(O)** Flow cytometry was performed to determine cell apoptosis after overexpressing circEGFR in MCF-7 and T47D cells. **(P)** Quantitative analysis of cell apoptosis in Figure O. Data are shown with the means ± SD. *, *P* < 0.05; **, *P* < 0.01; ***, *P* < 0.001.


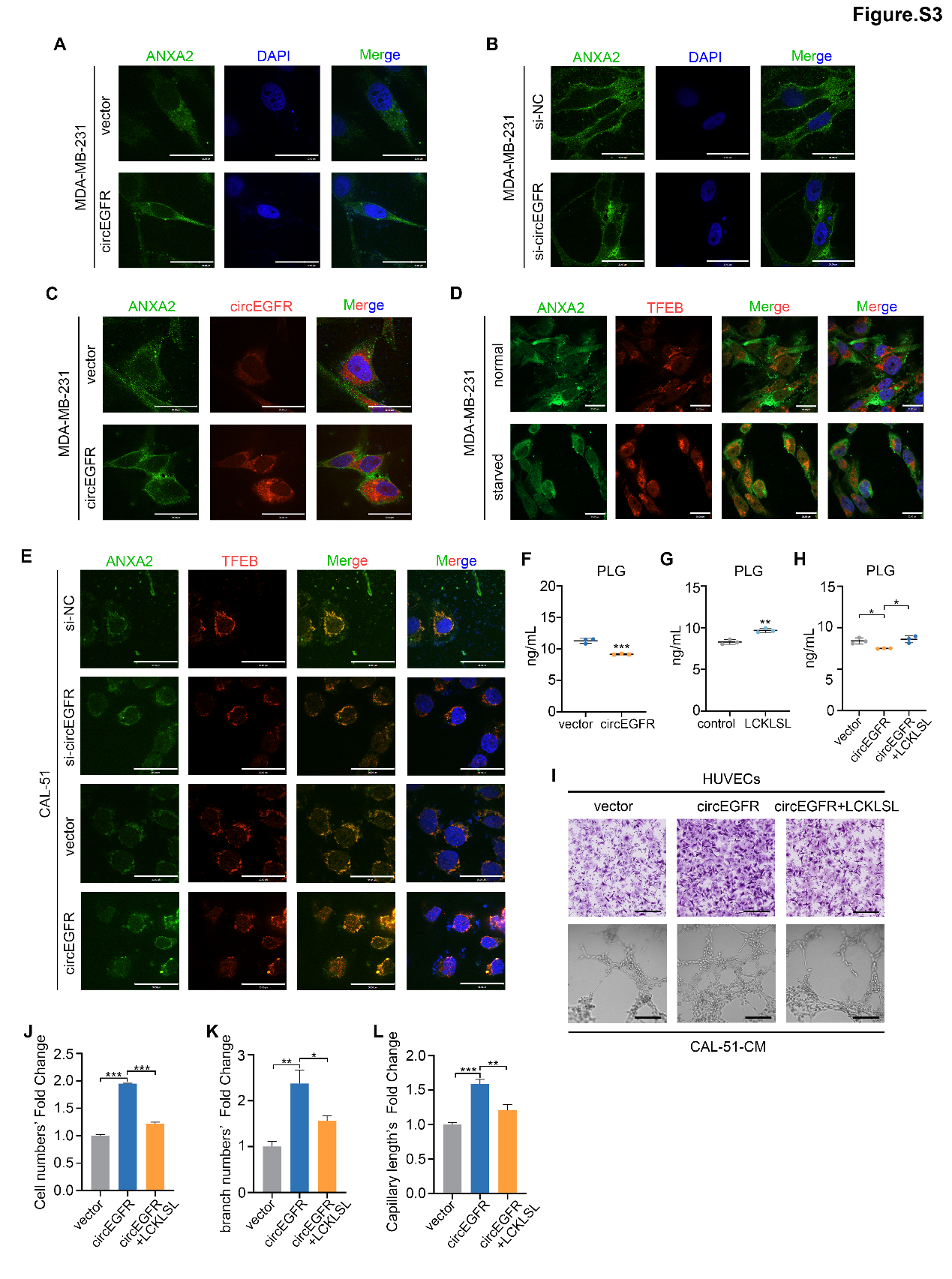


**Fig. S3 circEGFR-regulated ANXA2 plasma membrane trafficking and TFEB nucleus translocation facilitate autophagy in TNBC. (A)** IF was performed in MDA-MB-231 cells expressing circEGFR or vector using ANXA2 antibody. Scale bar, 30 μm. **(B)** IF was performed in MDA-MB-231 cells transfected with si-circEGFR or si-NC using ANXA2 antibody. Scale bar, 30 μm. **(C)** IF and FISH were conducted in MDA-MB-231 cells expressing circEGFR or vector using ANXA2 antibody and circEGFR probe. Scale bar, 30 μm. **(D)** MDA-MB-231 cells were cultured with the amino acid-free DMEM (starved) for 48 h, and then IF was performed using ANXA2 and TFEB antibodies. Scale bar, 30 μm. **(E)** IF was performed in CAL-51 cells knocked down or overexpressed with circEGFR using ANXA2 and TFEB antibodies. Scale bar, 30 μm. **(F)** The levels of PLG were measured in MDA-MB-231 cells expressing circEGFR or vector by ELISA. **(G)** The levels of PLG were measured in MDA-MB-231 cells treated with or without LCKLSL by ELISA. **(H)** The levels of PLG were measured in MDA-MB-231 cells expressing circEGFR or vector and treated with or without LCKLSL by ELISA. **(I)** Representative images of HUVECs cultured with conditioned medium from CAL-51 cells overexpressing circEGFR and treated with or without LCKLSL were measured by migration assay (original magnification, ×100) and matrigel tube formation assay (scale bar: 2 mm). **(J)** Quantitative analysis of cell numbers in migration assay. **(K)** Quantitative analysis of branch numbers in matrigel tube formation assay. **(L)** Quantitative analysis of capillary length in matrigel tube formation assay. Data are shown with the means ± SD. *, *P* < 0.05; **, *P* < 0.01; ***, *P* < 0.001.


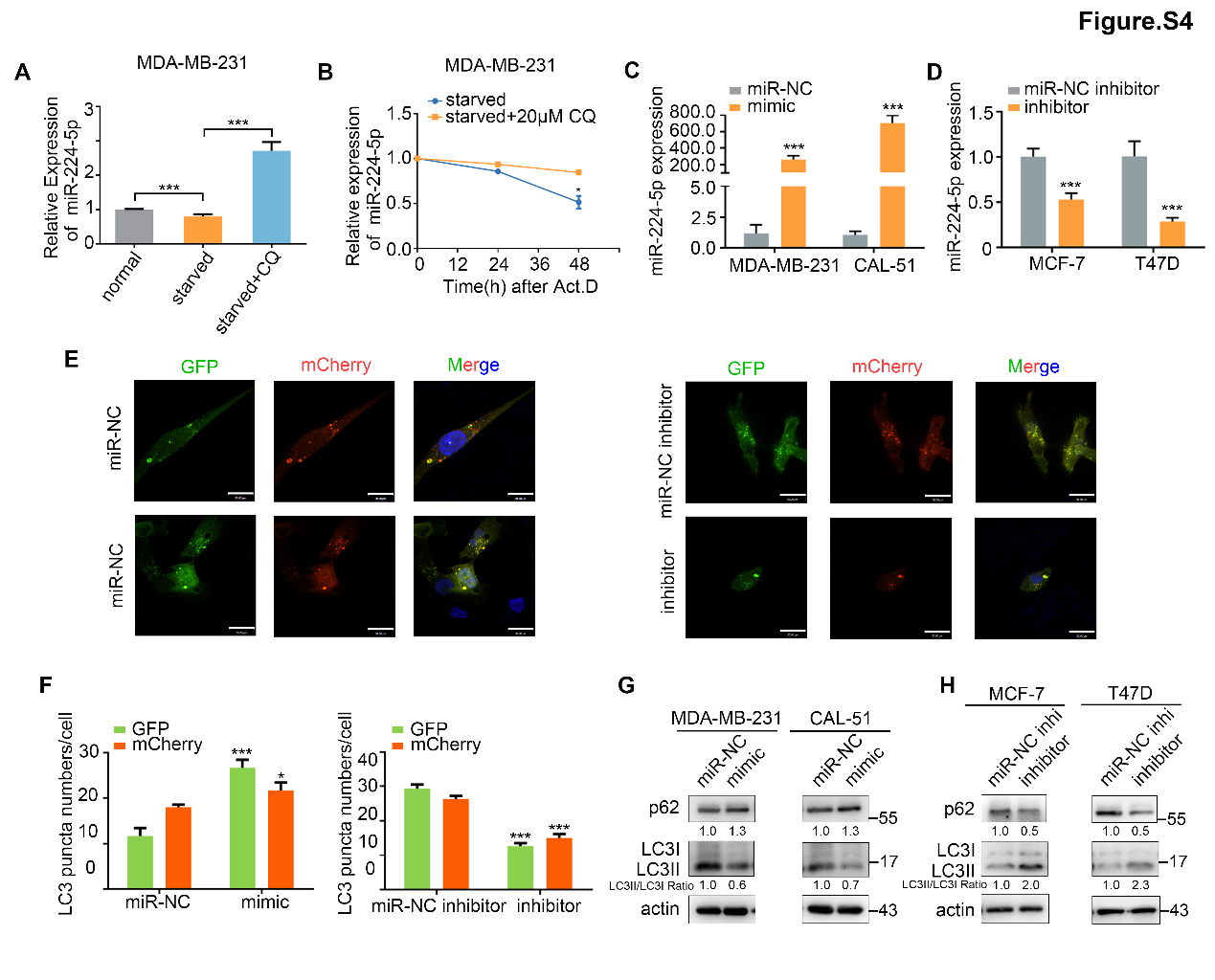


**Fig. S4 miR-224-5p is degraded during autophagic progression and inhibits autophagy process. (A)** The expression of miR-224-5p in normal, starved, and CQ-treated starved MDA-MB-231 cells was determined by qPCR. **(B)** qPCR analysis of miR-224-5p stability with or without actinomycin D treatment in starved MDA-MB-231 cells. **(C)** qPCR showed the expression of miR-224-5p in MDA-MB-231 and CAL-51 cells transfected with mimics or miR-NC. **(D)** qPCR showed the expression of miR-224-5p in MCF-7 and T47D cells transfected with inhibitors or miR-NC inhibitor. **(E)** Representative images of autophagy levels in GFP-mCherry-LC3B-labeled MDA-MB-231 cells transfected with miR-224-5p mimics or inhibitors were observed by IF. Scale bar, 30 μm. **(F)** Quantitative analysis of LC3 puncta numbers in Figure E. **(G)** Western Blot results of autophagy-related protein in MDA-MB-231 and CAL-51 cells transfected with miR-224-5p mimics. **(H)** Western Blot results of autophagy-related protein in MCF-7 and T47D cells transfected with miR-224-5p inhibitors. Data are shown with the means ± SD. *, *P* < 0.05; ***, *P* < 0.001.


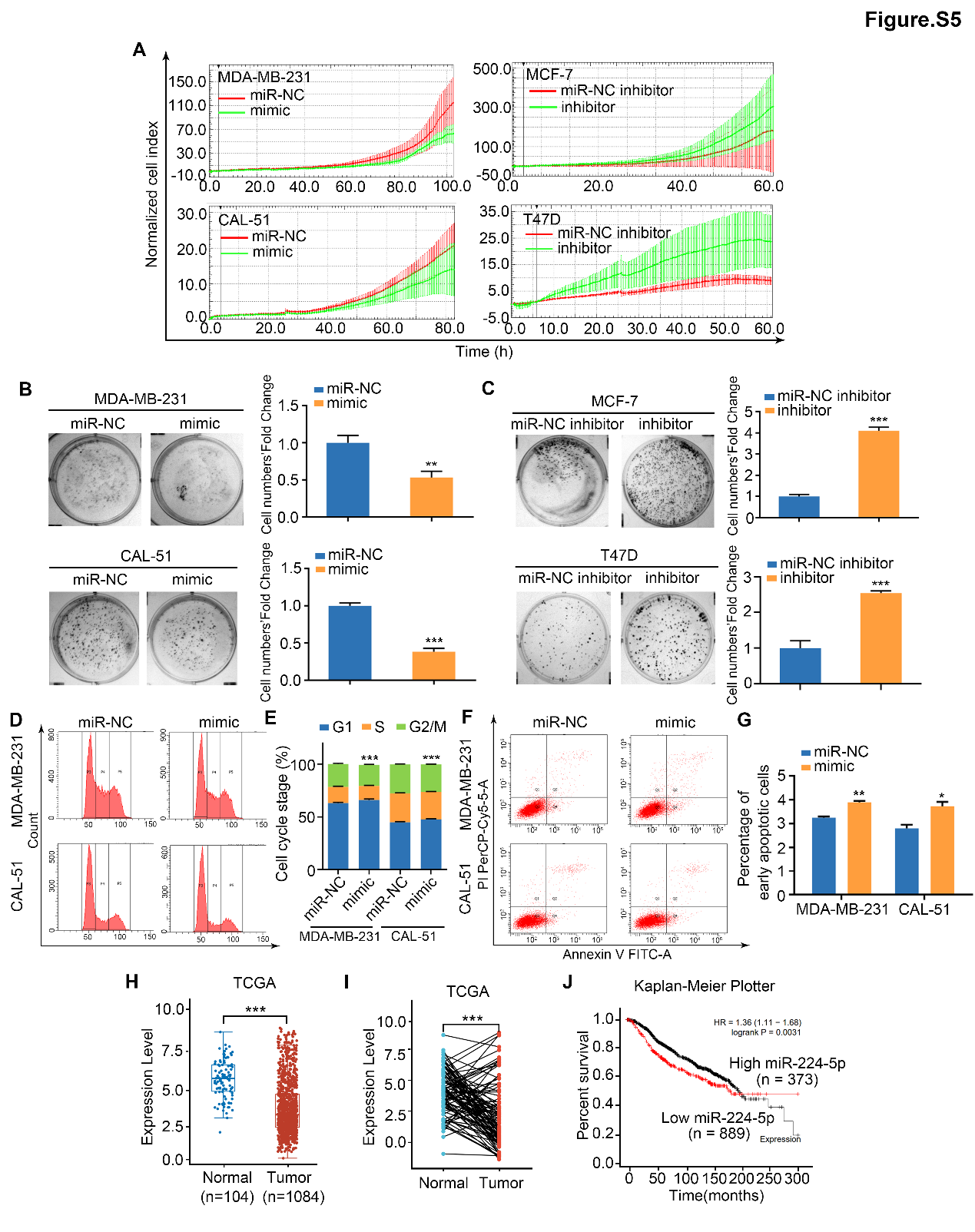


**Fig. S5 miR-224-5p inhibits malignant phenotypes of breast cancer cells. (A)** The xCELLigence RTCA-MP system was used to analyze cell proliferation in after overexpressing or knocking down miR-224-5p in BC cells. **(B)** Colony formation assay was performed to analyze on cell colony formation after overexpressing miR-224-5p in MDA-MB-231 and CAL-51 cells. **(C)** Colony formation assay was performed to analyze on cell colony formation after knocking down miR-224-5p in MCF-7 and T47D cells. **(D)** Flow cytometry was used to analyze cell cycle in MDA-MB-231 and CAL-51 cells transfected with miR-224-5p mimics or miR-NC. **(E)** Quantitative analysis of cell cycle in Figure D. **(F)** Flow cytometry was performed to determine cell apoptosis after overexpressing miR-224-5p in MDA-MB-231 and CAL-51 cells. **(G)** Quantitative analysis of cell apoptosis in Figure F. **(H)** The expression of miR-224-5p in normal and breast cancer tissues in the TCGA database. **(I)** The expression of miR-224-5p in breast cancer tissues and adjacent tissues in the TCGA database. **(J)** The relationship between survival and miR-224-5p levels in breast cancer patients by Kaplan-Meier plotter. Data are shown with the means ± SD. *, *P* < 0.05; **, *P* < 0.01; ***, *P* < 0.001.


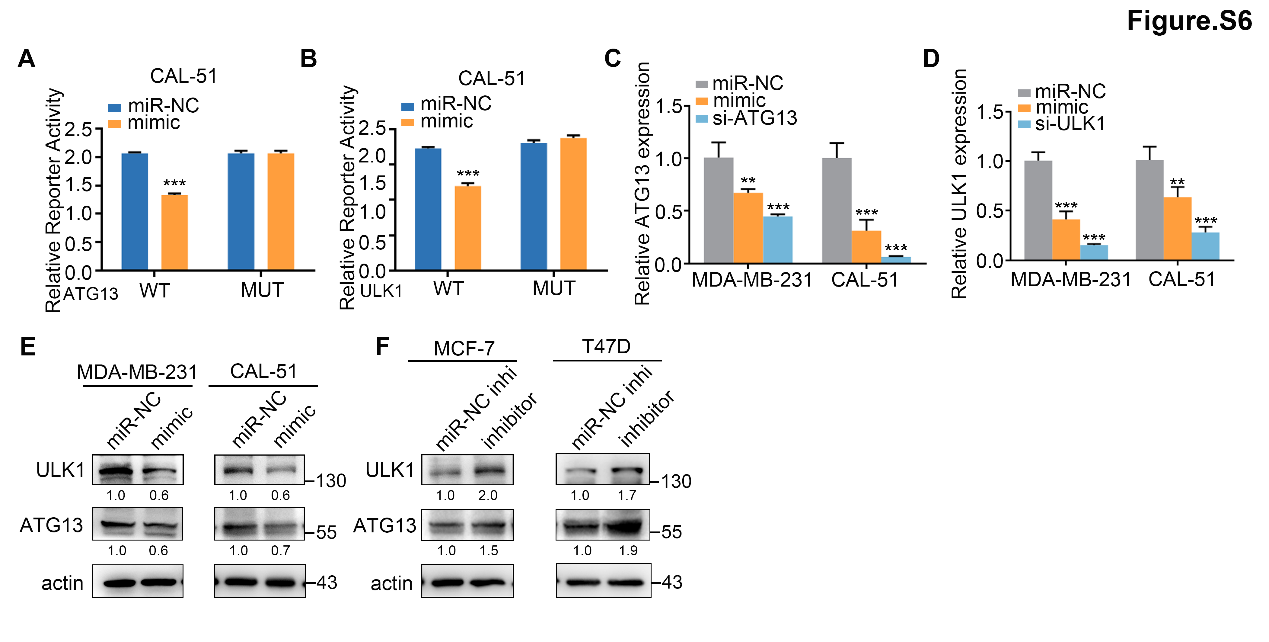


**Fig. S6 ATG13 and ULK1 are target genes of miR-224-5p. (A)** Luciferase activity was detected in CAL-51 cells co-transfected of ATG13-WT plasmid or ATG13-MUT plasmid and miR-224-5p mimics or miR-NC. **(B)** Luciferase activity was detected in CAL-51 cells co-transfected of ULK1-WT plasmid or ULK1-MUT plasmid and miR-224-5p mimics or miR-NC. **(C)** The expression of ATG13 in MDA-MB-231 and CAL-51 cells transfected with miR-NC, miR-224-5p mimics or si-ATG13 was measured by qPCR. **(D)** The expression of ULK1 in MDA-MB-231 and CAL-51 cells transfected with miR-NC, miR-224-5p mimics or si-ULK1 was measured by qPCR. **(E)** Western Blot results of ULK1 and ATG13 in MDA-MB-231 and CAL-51 cells transfected with miR-224-5p mimics or miR-NC. **(F)** Western Blot results of ULK1 and ATG13 in MCF-7 and T47D cells transfected with miR-224-5p inhibitors or miR-NC inhibitor. Data are shown with the means ± SD. **, *P* < 0.01; ***, *P* < 0.001.


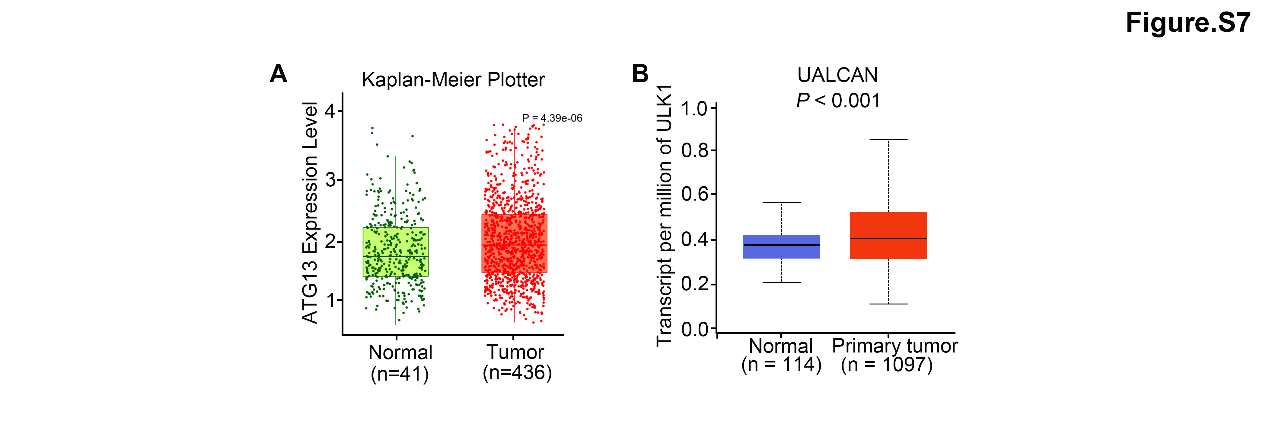


**Fig. S7 ATG13 and ULK1 are significantly higher expressed in breast cancer tissues. (A)** The expression of ATG13 in normal and breast cancer tissues by Kaplan-Meier plotter. **(B)** The expression of ULK1 in normal and primary tumor tissues by UALCAN.

**Supplementary Table**

**Table S1. Correlation between circEGFR expression and clinicopathologic characteristics of** **tissues from breast cancer patients**

| **Characteristics** | **No. of cases** | **circEGFR expression** | | |
| --- | --- | --- | --- | --- |
|  |  | **High** | **Low** | ***P*-value^i^** |
| **Age** |  |  |  | **0.050** |
| < 55 | 37 | 14 | 23 |  |
| ≥ 55 | 48 | 29 | 19 |  |
| **Stage** |  |  |  | **0.263** |
| I | 10 | 4 | 6 |  |
| II | 50 | 23 | 27 |  |
| III | 25 | 16 | 9 |  |
| **Lymphatic metastasis** |  |  |  | **0.034^*^** |
| Negative | 34 | 11 | 23 |  |
| Positive | 39 | 23 | 16 |  |
| **Tumor classification** |  |  |  |  |
| **Luminal** |  |  |  | **0.005^**^** |
| (+) | 45 | 29 | 16 |  |
| (−) | 40 | 13 | 27 |  |
| **Her2** |  |  |  | **0.483** |
| (+) | 9 | 6 | 3 |  |
| (−) | 76 | 37 | 39 |  |
| **TNBC** |  |  |  | **0.024^*^** |
| (+) | 31 | 21 | 10 |  |
| (−) | 54 | 22 | 32 |  |

^i^Chi-square test and Fisher’s exact test, *, *P* <0.05, **, *P* <0.01.

**Table S2. Correlation between circEGFR expression and clinicopathologic characteristics of plasma from breast cancer patients**

| **Characteristics** | **No. of cases** | **circEGFR expression** | | |
| --- | --- | --- | --- | --- |
|  |  | **High** | **Low** | ***P*-value^i^** |
| **Age** |  |  |  | **0.559** |
| < 55 | 57 | 31 | 26 |  |
| ≥ 55 | 15 | 10 | 5 |  |
| **Stage** |  |  |  | **0.829** |
| I | 6 | 4 | 2 |  |
| II | 52 | 28 | 24 |  |
| III | 14 | 8 | 6 |  |
| **Lymphatic metastasis** |  |  |  | **0.018^*^** |
| Negative | 36 | 15 | 21 |  |
| Positive | 36 | 23 | 13 |  |
| **Tumor classification** |  |  |  |  |
| **Luminal** |  |  |  |  |
| (+) | 58 | 28 | 30 | **0.145** |
| (−) | 14 | 10 | 4 |  |
| **Her2** |  |  |  |  |
| (+) | 9 | 6 | 3 | **0.140** |
| (−) | 63 | 22 | 41 |  |
| **TNBC** |  |  |  |  |
| (+) | 4 | 3 | 1 | **0.131** |
| (−) | 68 | 23 | 45 |  |

^i^Chi-square test and Fisher’s exact test, *, *P* <0.05.

**Table S3. List of nucleic acid sequences for qPCR Primers**

| **Gene** | **Sequence (5’-3’)** | **Application** |
| --- | --- | --- |
| circEGFR | F: ACCAAGCAACAAAATCCTGCAT  R: TCCATCGACATGTTGCTGAGA | qPCR |
| circEGFR | GCAGGATTTTGTTGCTTGGT | FISH |
| circEGFR | circEGFR probe: CGCGGCAGGACCAAGCAACAAAATCCTGCATGGCGCCGTG  NC probe:  CACGGCGCCATGCAGGATTTTGTTGCTTGGTCCTGCCGCG | RNA pulldown |
| EGFR | F: CCTGAGCTCTCTGAGTGCAAC  R: TGTTTCCAGACAAGCCACTCA | qPCR |
| U6 | F: CTCGCTTCGGCAGCACA  R: AACGCTTCACGAATTTGCGT | qPCR |
| GAPDH | F: TATGACAACAGCCTCAAGAT  R: AGTCCTTCCACGATACCA | qPCR |
| ATG13 | F: TCACTTTGTGGACCGTCCCTA  R: TGGTACACACTTCTTGAGAGTCT | qPCR |
| ULK1 | F: AGCACGATTTGGAGGTCGC  R: GCCACGATGTTTTCATGTTTCA | qPCR |
| miR-224-5p  sense | CAAGTCACTAGTGGTTCCGTTTA | qPCR |
| miRNA sense | CGCAAGTCACTAGTGGTTCCG | qPCR |
| miR-224-5p RT | GTCGTATCCAGTGCAGGGTCCGAGGTATTCGCACTGGATACGACtaaacg | qPCR |
| ATG2B | F: CAGACACGGCTCAGACCATT  R: GCACGTTTGACGTTGCTTCT | qPCR |
| ATG4A | F: TGGAATTGGCCCAGGATGAC  R: ATATGGCCGACTCAACGACC | qPCR |
| ATG5 | F: TCCCTCTTGGGGTACATGTCT  R: CGTCCAAACCACACATCTCG | qPCR |
| Gm13008 | F: GCTTTGTGGCCATTGTGCAT  R: CATTTGCCCGTCCCAATGTC | qPCR |
| ANXA2 | F: GGAGAGTTTCCCGCTTGGTT  R: GCACTTGGGGGTGTAGAGTG | qPCR |
| Convergent  Primer | F: GTTCAGCAACAACCCTGCC  R: CCCAGGTGGTTCTGGAAGTC | qPCR |
| si-circEGFR | ATGCAGGATTTTGTTGCTTGGT | si-RNA |
| si-ATG13 | GGAAAUUUGGUGUCUUGAAT | si-RNA |
| si-ULK1 | AAGGACCGCAUGGACUU | si-RNA |
| miR-224-5p  mimic | sense：  UCAAGUCACUAGUGGUUCCGUUUAG  antisense: CTAAACGGAACCACTAGTGACTTGA | si-RNA |
| miR-224-5p  inhibitor | UCAAGUCACUAGUGGUUCCGUUUAG | si-RNA |
| hsa_circ_0093887 | F: CCCTCAAAGTAAGACCAGTAGCA  R: GAGCTTGCATGTGAGGCTCT | qPCR |
| hsa_circ_0072100 | F: GTATCCTCCACCGTACAGCC  R: GCTACTGGAGCCTGATGGAC | qPCR |
| hsa_circ_0093890 | F: TGACCTCCTCATTGTTATTGGGT  R: GGAAGTCTACAGCAAGGCGA | qPCR |
| hsa_circ_0139982 | F: TATGAAGCTAGGCAGCCCCT  R: ATAACCAACCCGCTTTGCAC | qPCR |
| hsa_circ_0084933 | F: GGCTTATGAGTGGAACATGGC  R: ACCACTAGTAACCGCTGCAA | qPCR |
| hsa_circ_0005865 | F: AGACCCGACATTGCTTCAGA  R: CCGCTTACCAAAATGAGGCG | qPCR |
| hsa_circ_0078467 | F: GTGAGCTTCCCAATGGTCCC  R: TGACAATCACCTGCCCATCG | qPCR |
| hsa_circ_0059284 | F: GGACCCATCTTCCTGTGCTG  R: CTGGGAGGTGAGGAAGCAAT | qPCR |
| hsa_circ_0073524 | F: CAGGCAAATGAGATCATGTGGAA  R: GCCCCTTTCAACGCCAATAC | qPCR |
| hsa_circ_0002603 | F: ATATGGTGTGCAGGAGCCATC  R: AAGTCTTTAGGGACACAGGGA | qPCR |
| hsa_circ_0010059 | F: GTTGGCATATGGGGCTGGAA  R: CCCACTCCTTTGGCAATCCC | qPCR |
| hsa_circ_0008760 | F: CTGCCTTGCTGACAGGTGTA  R: TGACTCCAATAAAGCTGGTGCT | qPCR |
| hsa_circ_0077078 | F: GTAAAAACAGCAGCCCAAAGC  R: GTCCCATCTTTATCTGTATCTCCT | qPCR |
| hsa_circ_0087589 | F: TTCTGTTCTGGGAGCACACC  R: AGGCTGGCATTGGCATAGTT | qPCR |
| hsa_circ_0107539 | F: AAACCCCCGATAGCAGCAAC  R: TTGTCTTTTTCTGTTTCTTCTGGGC | qPCR |
| hsa_circ_0057569 | F: GCAAAGTCTTGTCCCCGTGT  R: TTAGGTGATTCCCAGGTCTGC | qPCR |
| hsa_circ_0005462 | F: TGGCACATCTCTTTGGAAGAAG  R: TGTCCATGACTCTGGGTTGC | qPCR |
| hsa_circ_0007918 | F: GCGTGGTAACCAGACCATTG  R: CGGCCTCTTAAATTGGGTGG | qPCR |
| hsa_circ_0011201 | F: GGCCCATTGTCATCCACTGC  R: CCTGCCCACCTCGACCA | qPCR |
| hsa_circ_0075060 | F: GAAGCCACACCTTTTCGGAAC  R: CAGCAGGTTTCCCCAGAGTG | qPCR |
| hsa_circ_0110042 | F: GAGAGCTGCCCTGACGAG  R: GAGATGACGGGTGGCTTTGA | qPCR |

**Table S4. Antibodies used in the experiments**

| **Product** | **Source** | **Dilution** | **No. of Catalogue** |
| --- | --- | --- | --- |
| ***Western Blot:*** |  |  |  |
| anti-p62 | Cell Signaling Technology | 1:1000 | 5114S |
| anti-LC3 | Sigma-Aldrich | 1:1000 | L8918 |
| anti-GAPDH | Proteintech | 1:50000 | 60004-1-Ig |
| anti-ULK1 | Proteintech | 1:1000 | 20986-1-AP |
| anti-ATG13 | Proteintech | 1:1000 | 18258-1-AP |
| anti-EGFR | Sigma-Aldrich | 1:1000 | E3138 |
| anti-ANXA2 | Proteintech | 1:10000 | 60051-1-Ig |
| anti-TFEB | Proteintech | 1:1000 | 13372-1-AP |
| anti-Lamin B | Santa Cruz | 1:1000 | sc-365962 |
| anti-MMP-9 | Santa Cruz | 1:1000 | sc-12759 |
| anti-VEGF | Santa Cruz | 1:1000 | sc-152 |
| anti-HSP70 | Proteintech | 1:1000 | 10995-1-AP |
| anti-Alix | Proteintech | 1:1000 | 12422-1-AP |
| anti-CD63 | Proteintech | 1:1000 | 25682-1-AP |
| anti-AGO2 | Proteintech | 1:1000 | 10686-1-AP |
| ***co-IP:*** |  |  |  |
| anti-ANXA2 | Proteintech | 1:10000 | 60051-1-Ig |
| anti-TFEB | Proteintech | 1:1000 | 13372-1-AP |
| anti-Mouse IgG | ZSGB-BIO | 1:5000 | ZB-2305 |
| anti-Rabbit IgG | ZSGB-BIO | 1:5000 | ZB-2301 |
| ***IHC:*** |  |  |  |
| anti-Ki67 | ZSGB-BIO | 1:100 | ZM-0166 |
| anti-ATG13 | Proteintech | 1:50 | 18258-1-AP |
| anti-ULK1 | Proteintech | 1:50 | 20986-1-AP |
| anti-vWF | Proteintech | 1:100 | 27186-1-AP |
| ***IF:*** |  |  |  |
| anti-ANXA2 | Proteintech | 1:500 | 60051-1-Ig |
| anti-TFEB | Proteintech | 1:100 | 13372-1-AP |
| DAPI | ZSGB-BIO | 1:10000 | ZLI-9557 |
| **Secondary antibody:** |  |  |  |
| ***Western Blot:*** |  |  |  |
| anti-rabbit IgG (H+L) | Promega | 1:3000 | W4011 |
| anti-mouse IgG (H+L) | Promega | 1:2000 | W4021 |
| ***IF:*** |  |  |  |
| Alexa Fluor® 594-labeled goat anti-Rabbit IgG (H+L) | ZSGB-BIO | 1:100 | ZF-0516 |
| FITC-labeled goat anti-Mouse IgG (H+L) | ZSGB-BIO | 1:100 | ZF-0312 |
